# Reconciliation and local gene tree rearrangement can be of mutual profit

**DOI:** 10.1186/1748-7188-8-12

**Published:** 2013-04-08

**Authors:** Thi Hau Nguyen, Vincent Ranwez, Stéphanie Pointet, Anne-Muriel Arigon Chifolleau, Jean-Philippe Doyon, Vincent Berry

**Affiliations:** 1 LIRMM, UMR 5506 CNRS - Université Montpellier 2, Montpellier Cédex 5, France; 2 Montpellier SupAgro (UMR AGAP), Montpellier, France; 3 Institut de Biologie Computationnelle, 95 rue de la Galéra, 34095 Montpellier cédex, France

**Keywords:** Evolution, Reconciliation, Gene Tree Correction, Method, Software, Duplication, Transfer, Loss, Nearest Neighbor Interchange

## Abstract

**Background:**

Reconciliation methods compare gene trees and species trees to recover evolutionary events such as duplications, transfers and losses explaining the history and composition of genomes. It is well-known that gene trees inferred from molecular sequences can be partly erroneous due to incorrect sequence alignments as well as phylogenetic reconstruction artifacts such as long branch attraction. In practice, this leads reconciliation methods to overestimate the number of evolutionary events. Several methods have been proposed to circumvent this problem, by collapsing the unsupported edges and then resolving the obtained multifurcating nodes, or by directly rearranging the binary gene trees. Yet these methods have been defined for models of evolution accounting only for duplications and losses, i.e. can not be applied to handle prokaryotic gene families.

**Results:**

We propose a reconciliation method accounting for gene duplications, losses and horizontal transfers, that specifically takes into account the uncertainties in gene trees by rearranging their weakly supported edges. Rearrangements are performed on edges having a low confidence value, and are accepted whenever they improve the reconciliation cost. We prove useful properties on the dynamic programming matrix used to compute reconciliations, which allows to speed-up the tree space exploration when rearrangements are generated by Nearest Neighbor Interchanges (NNI) edit operations. Experiments on synthetic data show that gene trees modified by such NNI rearrangements are closer to the correct simulated trees and lead to better event predictions on average. Experiments on real data demonstrate that the proposed method leads to a decrease in the reconciliation cost and the number of inferred events. Finally on a dataset of 30 *k* gene families, this reconciliation method shows a ranking of prokaryotic phyla by transfer rates identical to that proposed by a different approach dedicated to transfer detection [BMCBIOINF 11:324, 2010, PNAS 109(13):4962–4967, 2012].

**Conclusions:**

Prokaryotic gene trees can now be reconciled with their species phylogeny while accounting for the uncertainty of the gene tree. More accurate and more precise reconciliations are obtained with respect to previous parsimony algorithms not accounting for such uncertainties [LNCS 6398:93–108, 2010, BIOINF 28(12): i283–i291, 2012].

A software implementing the method is freely available at http://www.atgc-montpellier.fr/Mowgli/.

## Background

A phylogenetic tree or *phylogeny* is a tree depicting evolutionary relationships among biological entities that are believed to have a common ancestor. A gene family is a group of genes descending from a common ancestor, that retains similar sequences and often similar functions [1]. A species tree depicts the evolutionary history of a group of species, whereas a gene tree depicts the evolutionary history of a gene family. Gene trees often differ from the species tree due to family-specific evolutionary events such as gene duplications, gene losses and horizontal gene transfers. By comparing a gene tree with the species tree, reconciliation methods try to recover those major evolutionary events. Reconciliation is indeed the process of constructing a mapping between a gene tree and a species tree to explain their differences and similitudes with evolutionary events such as speciation (), duplication (), loss (), and horizontal gene transfer () events. Reconciliations are most often inferred on the basis of a parsimony criterion: a cost is given to each event type, the total cost of a reconciliation is the sum of the costs of the individual events it uses, and a reconciliation of minimum total cost is sought for. This computational problem is often called *Most Parsimonious Reconciliation*, or MPR in short, and many works have been devoted to it recently [2-8].

The first proposed models focused on parsimonious reconciliations involving only duplications and losses (the DL model) [9-11] or only horizontal transfers and losses [12]. Probabilistic methods have also been developed for the DL model, such as that of Arvestad et al. [13] (see Doyon et al. [14] for a review). Most recent works using a parsimony approach have been devoted to models incorporating duplications, losses and transfers all together (the DTL model) [2,4,5,8], which is necessary to handle prokaryotes. When accounting for transfer events, the history proposed by a reconciliation is consistent if, for any transfer, the donor and receiver species co-exist. Ensuring such a time consistency is difficult and leads to an NP-hard problem in the general case [7,15] which cannot be solved by just examining couples of species tree edges. However, in the case divergence dates are available for nodes of the species tree, the problem becomes amenable [2,16]. The difficulty to handle transfers has led to a split within proposed DTL methods, namely those that ensure time-consistency [2,16] and those that do not [3,4,7]. The fastest parsimony algorithms for the later category runs in *O*(*mn* log*n*) where *m* and *n* are the sizes of the gene and species trees respectively [3], while the fastest time-consistent algorithm runs in *O*(*mn*^2^) [2]. Probabilistic methods also have been extended recently to the DTL model. Inspired by the work of Tofigh [17], Szőllösi et al. recently proposed a time-consistent procedure to estimate the species tree by reconciliations from a set of gene trees [18].

A major problem, when applying reconciliation methods, is that parts of the gene trees can be incorrect. This leads reconciliation methods to overestimate (), (), () and () events [19,20]. Errors within a binary gene tree can be due to sequence alignment problems, phylogenetic reconstruction artifacts (e.g. long branch attraction) or a lack of phylogenetic signal (especially for genes encoded by short sequences). Such phenomena are well-known in phylogenetics and several support measures, such as bootstrap values or bayesian posterior probabilities, have been proposed to detect unreliable edges in a gene tree. Up to now, very few works have tackled the reconciliation problem in the presence of unsupported edges, and most of them consider only the DL model [19,21-26]. Durand et al. proposed an exponential exact algorithm to find the best rearrangement of a gene tree while preserving its strongly supported edges [19]. Another approach is to collapse unsupported edges, thereby creating nodes with more than two children (i.e., *polytomies*), and then to rely on a generalization of the least common ancestor mapping (LCA) to avoid the need for examining all possible binary rearrangements of the polytomies [21-23,26]. In this way, Chang et al. and Lafond et al. proposed polynomial time algorithms to solve the MPR problem for a binary species tree and a non-binary gene tree [22,26]. When both the species tree and the gene tree are non-binary, Berglund et al. proved that finding a refinement of the gene tree using less than a given number of duplications is an NP-complete problem [21]. They also proposed a heuristic approach to refine the gene tree by first minimizing duplications and then losses. Zheng et al. showed that minimizing together duplication and loss costs is NP-hard for reconciling a non-binary species tree with a binary gene tree [25]. For this specific case, Vernot et al. proposed a fixed parameter tractable (FPT) algorithm whose complexity is exponential only in the maximum degree of nodes [23]. More recently, Stolzer et al. extended this FPT algorithm by allowing transfers [27].

Overall, several works relied on tree edit operations to deal with uncertainties in the gene trees. Durand et al. used Nearest Neighbor Interchange (NNI) edit operations to rearrange the local topology of the gene trees in the regions of low supports [19]. Górecki and Eulenstein proposed an efficient algorithm to do a similar task and at the same time root the gene trees, while restraining their search to trees that are at most *k* NNI moves away from the original gene trees [28]. Chaudhary et al. investigated Subtree Prune and Regraft (SPR) and Tree Bisection and Reconstruction (TBR) edit operations to search for the gene tree rearrangement that minimizes the number of duplications, regardless of losses [24].

It seems hard to have an exact polynomial time algorithm for the MPR problem under the DTL model even when the polytomies are present only in the gene tree or in the species tree. Following the works cited above to deal with uncertainties in the gene trees, we propose a heuristic method relying on NNI edit operations to search for a gene tree rearrangement that preserves strongly supported edges and minimizes the cost of reconciliation to a fixed binary species tree, but in the context of the more complex DTL model. The resulting dynamic program, called *MowgliNNI*, is a generalization of *Mowgli*[2], a program initially developed for fixed binary gene trees.

Experiments on simulated data show that *MowgliNNI* provides a gene tree that is closer to the true evolutionary history of the gene family, and leads to more accurate ,  and  predictions. Experiments on real data show a significant decrease in the number of predicted events and an increased precision, that is a decrease in the number of equally most parsimonious reconciliations. We conducted a large scale experiment where 30 k prokaryotic gene families covering several phyla were reconciled using *MowgliNNI*. These phyla were then ordered according to their inferred transfer rate. We obtained the same phyla ordering as the one obtained using Prunier, a method dedicated to transfer prediction [29,30], and our reconciliation based approach has the advantage of providing extra information: explicit donor and receiver branches for transfers, prediction and localization of duplications and losses.

## Methods

### Basic notations

Trees considered in this paper are rooted and labeled at their leaves only, each leaf being labeled with the name of a studied species. Given a tree *T*, its node set, edge set, leaf node set and root are resp. denoted *V*(*T*), *E*(*T*), *L*(*T*) and *r*(*T*). The label of a leaf *u* of *T* is denoted by ℒ(u) and the set of labels of leaves of *T* is denoted by ℒ(T). When a node *u* has two children, they are denoted *u*_1_ and *u*_2_.

Given two nodes *u* and *v* of *T*, we write *u* ≤ _*T*_*v* (resp. *u* < _*T*_*v*) if and only if *v* is on the unique path from *u* to *r*(*T*) (resp. and *u* ≠ *v*); if neither *u* < _*T*_*v* nor *v* < _*T*_*u* then *u* and *v* are said to be *incomparable*. As we consider rooted trees *T* only, we adopt the convention that an edge denoted (*v*,*u*) means that *u* < _*T*_*v*. For a node *u* of *T*, *T*_*u*_ denotes the subtree of *T* rooted at *u*, *p*(*u*) the parent node of *u*, while (*u*_*p*_,*u*) is the *parent edge* of *u*. A tree *T*^′^ is a *refinement* of a tree *T* if *T* can be obtained from *T*^′^ by collapsing some edges in *T*^′^, *i.e.* by merging the two extremitites of these edges [31].

A *species tree* is a rooted binary tree depicting the evolutionary relationships of ancestral (internal nodes) species leading to a set of extant (leaf) species. A species tree *S* is considered here to be *dated*, that is associated to a time function $\theta_{S}:V(S)\to R^{+}$ such that if *y* < _*S*_*x* then *θ* (*y*) < *θ* (*x*). Such times are usually estimated on the basis of molecular sequences [32] and fossil records. Note that to ensure the time consistency of inferred transfers, absolute dates are not required, the important information being the ordering of the nodes of *S* induced by the dating.

**Figure 1:**
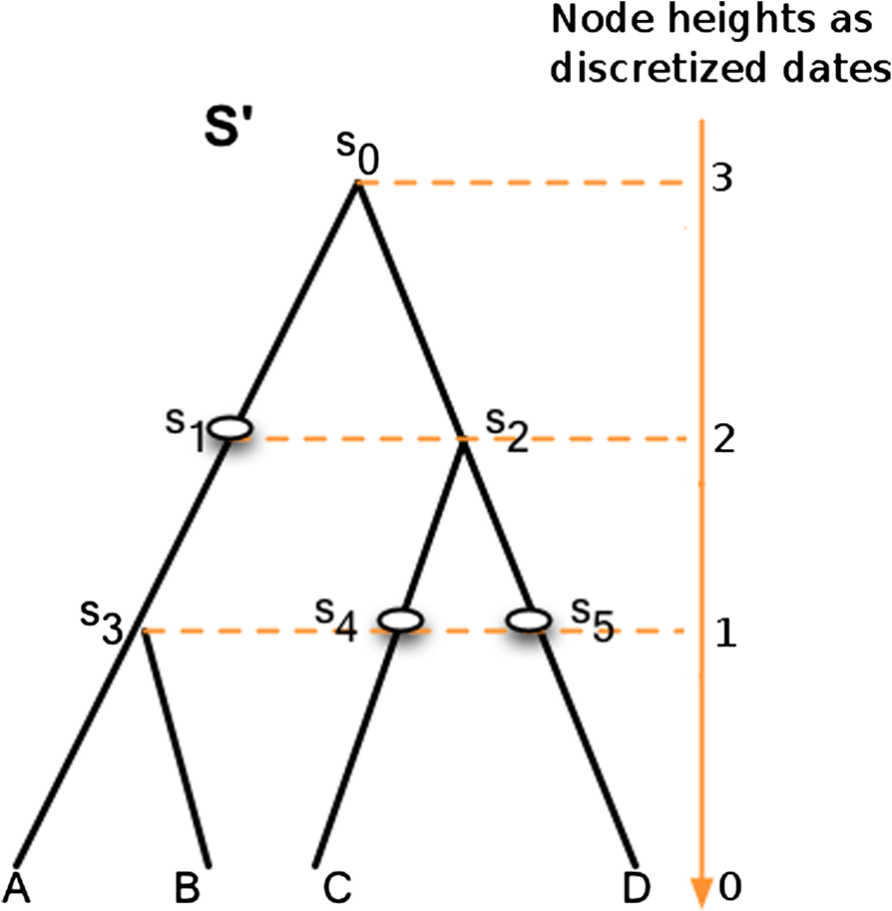
**Example of subdivision of a species tree.** The *subdivision* ***S***^***′***^ is obtained from the species tree ***S*** by splitting some of its edges thanks to additional *artificial* nodes (***∘***), i.e. nodes with a single child. These nodes are added on edges at the precise date a node appears somewhere else in *S*. For instance here, the artificial node *s*_1_ is placed at the same date as the node *s*_2_ of *S*, while *s*_4_ and *s*_5_ are placed at the same date as *s*_3_.

Given a dated binary species tree *S*, the reconciliation model we rely on considers a variant *S*^′^ of *S* called a *subdivision* (as done also in [2,6,17]), associated to a time function $\theta_{S^{\prime }}$. More precisely, for each node *x* ∈ *V*(*S*) ∖ *L*(*S*) and each edge (*y*_*p*_,*y*) ∈ *E*(*S*) s.t.  *θ*_*S*_(*y*_*p*_) > *θ*_*S*_(*x*) > *θ*_*S*_(*y*), an *artificial* node *w* is inserted along the edge (*y*_*p*_,*y*), with $\theta_{S^{\prime }}(w)=\theta_{S}(x)$ (see Figure 1). Note that the height of *S*^′^ nodes (i.e. the number of their ancestors) is a valid time function that preserves the same partial order on nodes as $\theta_{S^{\prime }}$ and that the restriction of this time function to *V*(*S*)⊆*V*(*S*^′^) preserves the partial order induced by *θ*_*S*_.

A *gene tree* is a rooted binary tree depicting the evolutionary history of a gene family, that lead to a set of homologous sequences observed in current organisms. Each leaf of the gene tree has a unique label, corresponding to specific extant sequences of the gene. Indeed, several leaves of a gene tree can be associated to a same species due to duplication and transfert events. We denote by *s*(*u*) the species associated to leaf *u* ∈ *V*(*G*).

A gene tree *Gwith supports* is a gene tree whose *internal* edges each have a support value. Let *wk*_*t*_(*G*) ⊆ *E*(*G*) be the set of edges having a support value weaker than threshold *t* and let *str*_*t*_(*G*) be *E*(*G*) - *wk*_*t*_(*G*), that is the edges having a support equal or stronger than *t*.

### Reconciliation model

Reconciling a gene tree *G* with a species tree *S* means building a mapping *α* that associates each gene *g* ∈ *V*(*G*) to a sequence of nodes in *V*(*S*), namely the species in which the sequence *g* evolved. This evolution is submitted to different kinds of biological events such as speciation, duplication and transfer. The following definition presents a discrete models of this evolution.

#### 

**Definition 1** (Reconciliation model). Consider a gene tree *G*, a species tree *S* with a time function *θ*_*S*_, and its subdivision *S*^′^ with a time function *θ*_*S*′_.

Let *α* be a function that maps each node *u* of *G* onto an ordered sequence of nodes of *S*^′^, denoted *α*(*u*). For *u* ∈ *V*(*G*), let *ℓ* denote the length of *α*(*u*) and let *α*_*i*_(*u*) be its *i*^th^ element (where 1 ≤ *i* ≤ *ℓ*). *α* is said to be a *reconciliation* between *G* and *S*^′^ if and only if exactly one of the following *atomic* events occurs for each couple of nodes *u* of *G* and *α*_*i*_(*u*) of *S*^′^ (where *α*_*i*_(*u*) is denoted *x*): 

• *x* is the last vertex *α*_*ℓ*_(*u*) and exactly one of the cases below is true. 

1. *u*∈*L*(*G*), *x* ∈ *L*(*S*^′^) and ℒ(x)=s(u). ( event)

2. *x* is not artificial and {*α*_1_(*u*_1_),*α*_1_(*u*_2_)} = {*x*_1_,*x*_2_}. ( event)

3. *α*_1_(*u*_1_)=*α*_1_(*u*_2_) = *x*. ( event)

4. *α*_1_(*u*_1_) = *x*, and *α*_1_(*u*_2_) = *x*^′^ is such that *x*^′^ ≠ *x* and $\theta_{S^{\prime }}(x^{\prime })=\theta_{S^{\prime }}(x)$. ( event)

• otherwise, one of the cases below is true. 

5. *x* is an artificial vertex and *α*_*i*+1_(*u*) is its only

child. ( event)

6. *x* is not artificial and *α*_*i*+1_(*u*) ∈ {*x*_1_,*x*_2_}. (SL event)

7. *α*_*i*+1_(*u*) = *x*^′^ is such that *x*^′^ ≠ *x* and $\theta_{S^{\prime }}(x^{\prime })=\theta_{S^{\prime }}(x)$. (TL event)

**Figure 2:**
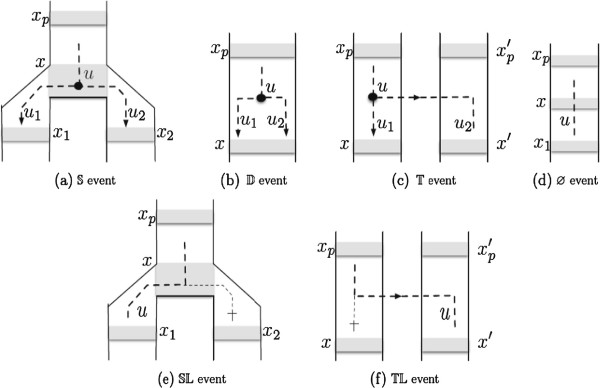
**The events of the reconciliation DTL model (Definition 1).** Each possible event is displayed for a node ***u*** of ***G*** and a node ***x*** of the subdivided species tree ***S***^***′***^ on which ***u*** is mapped. Note that a same node *u* can be mapped to several nodes. As a result of the mapping of its nodes, the gene tree *G*, extended here with losses induced by the mapping (‡), is embedded in *S*^′^ (here dashed lines represent edges of *G*, and plain lines those of *S*^′^, grayed rectangular zones represent nodes of *S*^′^).

**Figure 3:**
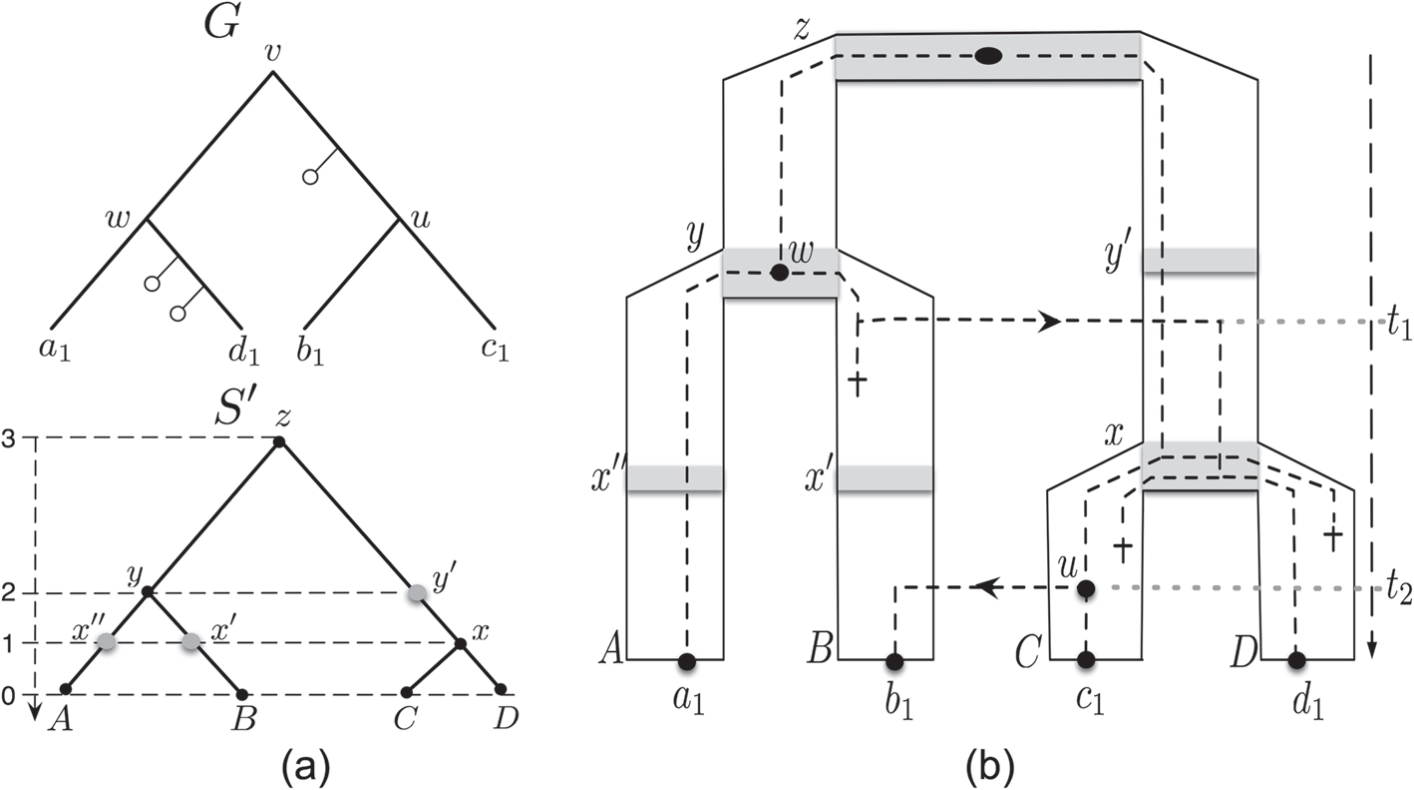
**Example of a DTL reconciliation.** (a) A gene tree ***G***, represented with lost copies of the gene (***∘***), and a subdivided species tree ***S***^***′***^. (**b**) A reconciliation *α* between *G* and *S*^′^. The reconciliation maps each node of *G* onto a sequence of nodes in *S*^′^, inducing evolutionary events. For instance, nodes *w*,*d*_1_ and *u* from *G* are mapped as follows: *α*(*w*) = [*y*], where *α*_1_(*w*) = *y* is an  event; *α*(*d*_1_) = [*x*^′^,*x*,*D*], where *α*_1_(*d*_1_) = *x*^′^ is a TL event, *α*_2_(*d*_1_) = *x* is an SL event, and *α*_3_(*d*_1_) = *D* is a  event; *α*(*u*) = [*y*^′^,*x*,*C*], where *α*_1_(*u*) = *y*^′^, *α*_2_(*u*) = *x*, and *α*_3_(*u*) = *C* are respectively a , an SL, and a  event.

The combinatorial events mentioned above (, , , , , TL, SL) are those defined in [2]. See Figure 2 for an illustration of these events and Figure 3 for an example of reconciliation according to this model.

Note that among these *events*, TL and SL are in fact a combination of two independent biological events. However, the fact that a loss is always taken into account jointly with another event allows to obtain a recursive algorithm and is done without loss of generality, i.e. does not reduce the power of the model [2].

Given a gene tree *G* and species tree *S*, there is an infinite number of possible reconciliations. Discrete evolutionary models compare them by counting the number of events they respectively induce. As different types of event can have different expectancies (*e.g.* are thought to be more frequent than  and ), reconciliation models allow for a specific *cost* to be given to each kind of event. The cost of a reconciliation is then the sum of the costs of the individual events it induces. In that setting, the parsimony approach is then to prefer a reconciliation of lower cost. This is formalized in the following definition.

#### 

**Definition 2.** Let us consider a gene tree *G*, a subdivision *S*^′^ of a species tree, and a reconciliation *α* between trees *G* and *S*^′^. The *cost* of *α* is defined as

cost(α)=dδ+tτ+lλ,

where *δ*, *τ*, and *λ* respectively denote the cost of , , and  events, while *d*, *t*, and *l* denote the number of the corresponding events in *α*. Moreover, a TL event is atomic and costs (*τ* + *λ*), while a SL event just costs *λ*. Indeed, speciation events are most of the time considered as having a null cost, but the model easily accommodates for non-null costs if necessary.

The *optimal reconciliation cost* is

$$C(G,S^{\prime })=\underset{\alpha }{\text{min}}\{\text{cost}(\alpha )\}$$

over all reconciliations *α* between *G* and *S*^′^.

Note that several distinct alternative reconciliations can have an optimal reconciliation cost.

#### **Lemma 1** (Consecutive TL events)

Consider a gene tree *G*, the subdivision *S*^′^ of a species tree, and a reconciliation *α* of optimal cost *C*(*G*,*S*^′^) = *c*(*α*). For any node *u* of *G*, if *α*_*i*_(*u*) corresponds to a TL event, then *α*_*i* + 1_(*u*) does not.

This results from the observation that two TL in a row can be replaced by single TL, leading to a reconciliation of lesser cost.

### Finding a most parsimonious reconciliation

To find one of the most parsimonious reconciliations between a gene *G* and a species tree *S* we will rely on the dynamic programming algorithm of Doyon et al. [2] that computes the optimal reconciliation cost, *C*(*G*,*S*^′^) on *G* and the subdivision *S*^′^ of *S*. This algorithm successively examines the nodes *u* of *G* and their possible mapping on nodes *x* of *S*^′^ (or equivalently on edges ending at such nodes). A node *u* of *G* can be mapped on such a vertex *x* according to different scenarios, each postulating a different event at node *u* among those of Definition 1. The optimal cost for mapping *u* at *x* is defined according to the scenario of minimal cost. For running time optimization reasons, the scenario involving a TL event, whose cost is denoted $c_{TL}(u,x)$, is computed after the other possible scenarios, $c_{\bar{TL}}(u,x)$ denoting the minimum cost that can be achieved among the latter. This decomposition is possible since a TL event is always followed by a , , , , , or SL event (see Lemma 1). As a result, the best receiver for a TL event of node *u* with donor branch *x* can be computed from the costs $c_{\bar{TL}}(u,y)$ over all vertices *y* other than *x* such that $\theta_{S^{\prime }}(y)=\theta_{S^{\prime }}(x)$. The cost $c_{\bar{TL}}(u,y)$ are themselves computed from $c_{TL}(u_{i},x)$ values but for children *u*_*i*_ of *u* (see below). These intricate notions are formally detailed in Definition 3

#### **Definition 3** (Reconciliation cost matrix).

Consider a gene tree *G* and the subdivision *S*^′^ of a species tree *S* with a time function *θ*_*S*′_. Let $c\;:V(G)\times V(S^{\prime })\to R$ denote the cost matrix recursively defined as follows for a node *u* of *G* and a vertex *x* of *S*^′^: $c_{\bar{TL}}(u,x)=\text{min}\{c_{E}(u,x)\;:E\in \{C,S,D,T,\emptyset ,SL\}\}$ and $c(u,x)=\text{min}\{c_{TL}(u,x),c_{\bar{TL}}(u,x)\}$, where the costs $c_{E}(u,x)$ for all events x E∈{C,S,D,T,∅,SL,TL} are defined below 

• $c_{C}(u,x)=0$, if *u* ∈ *L*(*G*), *x* ∈ *L*(*S*^′^) and ℒ(x)=s(u).

• $c_{S}(u,x)=\text{min}\{c(u_{1},x_{1})+c(u_{2},x_{2}),$

• *c*(*u*_1_,*x*_2_) + *c*(*u*_2_,*x*_1_)}, if *u* ∉ *L*(*G*) and $x\notin L(S^{\prime })$.

• $c_{D}(u,x)=c(u_{1},x)+c(u_{2},x)+\delta$, if *u* ∉ *L*(*G*).

• $c_{T}(u,x)=\text{min}\{c(u_{1},x)+c(u_{2},z),c(u_{1},y)+c(u_{2},x)\}$

• + *τ*, with *u* ∉ *L*(*G*) and *z* (resp. *y*) denoting a vertex that minimizes *c*(*u*_2_,*z*) (resp. *c*(*u*_1_,*y*)) over all vertices $x^{\prime }\in V(S^{\prime })\setminus \{x\}$ such that $\theta_{S^{\prime }}(x^{\prime })=\theta_{S^{\prime }}(x)$.

• $c_{\emptyset }(u,x)=c(u,x_{1})$, if *x* has a single child.

• $c_{SL}(u,x)=\text{min}\{c(u,x_{1}),c(u,x_{2})\}+\lambda$, if *x* has two children.

• $c_{TL}(u,x)=c_{\bar{TL}}(u,y)+\tau +\lambda$, where *y* denotes a vertex that minimizes $c_{\bar{TL}}(u,y)$ over all vertices *x*^′^ ∈ *V*(*S*^′^) ∖ {*x*} such that $\theta_{S^{\prime }}(x^{\prime })=\theta_{S^{\prime }}(x)$.

If the above constraints for an event E∈{C,S,D,T,∅,SL,TL} on node *u* and vertex *x* are not respected, the corresponding cost $c_{E}(u,x)$ is set to *∞*.

The value *c*(*u*,*x*) is the optimal cost when mapping gene node *u* to node *x* in *S*^′^. The optimal cost for reconciling *G* with *S*^′^, denoted *C*(*G*,*S*^′^), is then $\text{mi}n_{x\in V(S^{\prime })}(c(r(G),x)$.

The algorithm of Doyon et al. [2], called *Mowgli*, fills the dynamic programming cost matrix $V(S^{\prime })\times V(G)\to R^{+}$ by two embedded loops: one loop visits all species nodes of *S*^′^ in time order (e.g. according to the $\theta_{S^{\prime }}$ partial order, while the other loop visits nodes of the gene tree *G* in postorder. Due to an optimization in precomputing the best receiver edge for transfer events of nodes *u* at a given time, this algorithm has *O*(|*S*|^2^.|*G*|) time complexity.

The problem considered in this paper is the following:

MOST PARSIMONIOUS RECONCILIATION GENE TREE (MPR-GT)

INPUT: 

• A dated species tree *S* with a time function *θ*_*S*_

• a gene tree *G* with supports on its edges and whose leaves are associated to leaves of *S*

• costs *δ*, *τ*, resp. *λ* for , , resp.  and

• a threshold *t*.

OUTPUT: a gene tree *G*^′^ such that both $ℒ(G)=ℒ(G^{*})$ and $\text{st}r_{t}(G)\subseteq E(G^{*})$, and such that *C*(*G*^∗^,*S*^′^) is minimum among all such trees.

### Algorithm

We describe here a heuristic for the MPR-GT problem that relies on a hill-climbing strategy to seek a (rooted) gene tree *G* of minimum reconciliation cost (see Definition 3) using NNI edit operations [33].

**Figure 4:**
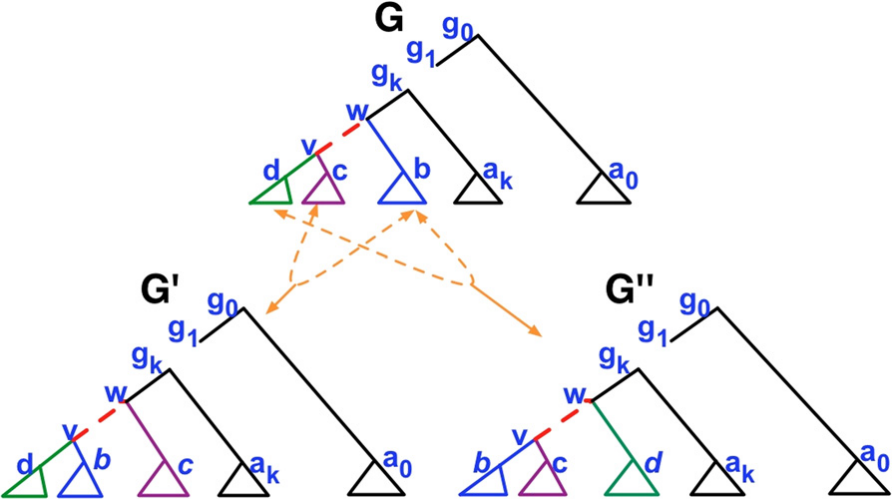
**A gene tree *G *with a weak edge*(w,v) *selected for an NNI.***v* is connected to two subtrees *G*_*c*_ and *G*_*d*_, while *w* is connected to *v* and to the subtree *G*_*b*_. Performing an NNI operation around (*w*,*v*) means interchanging subtree *G*_*b*_ with either *G*_*c*_ or *G*_*d*_, leading to trees $G^{\prime }$ and  respectively.

**Figure 5:**
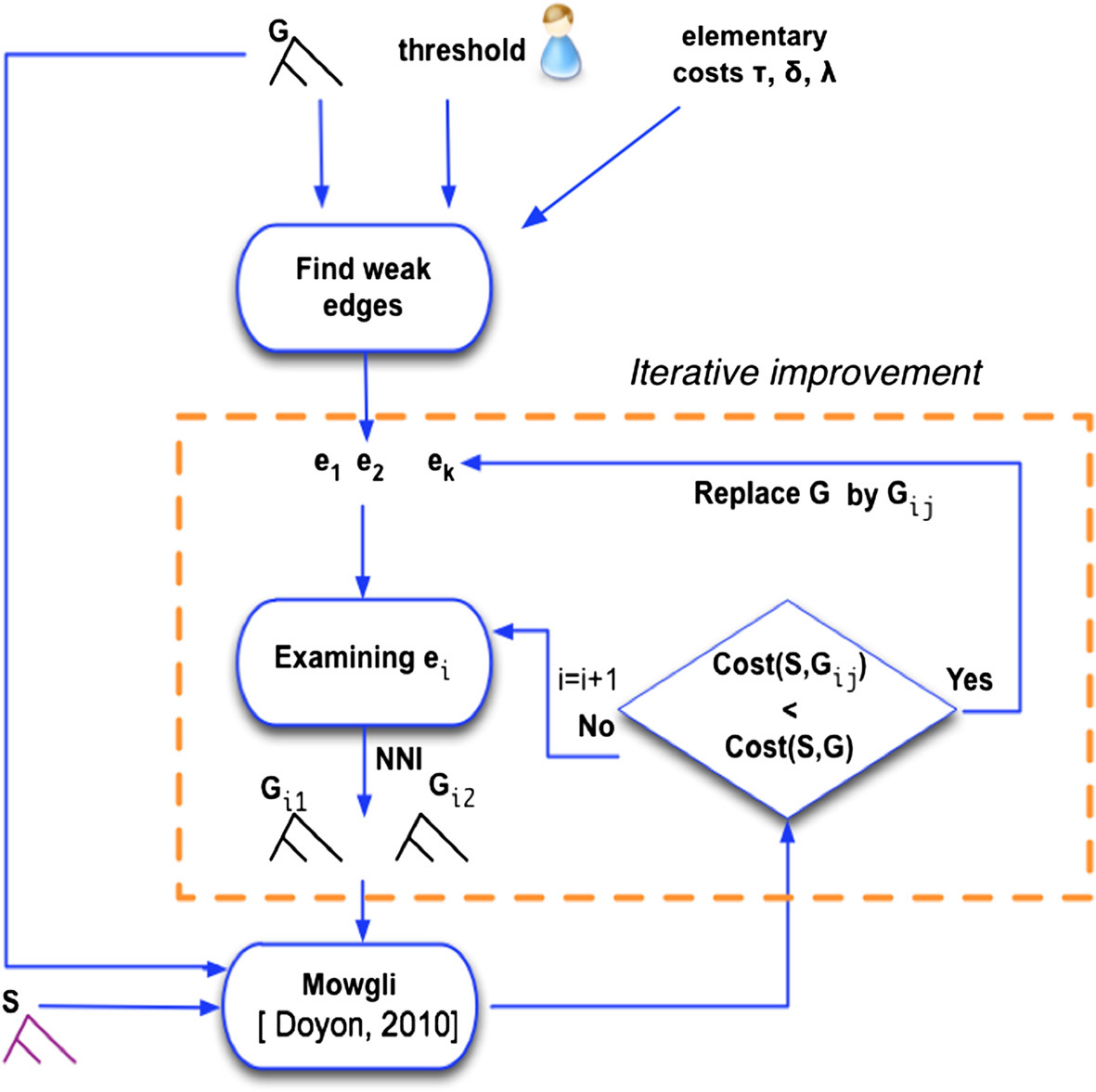
**Algorithmic scheme of *MowgliNNI***^***n***^** (non-optimized version of the method).**

Performing an NNI operation around an *internal* edge (*w*,*v*) means swapping the position of one of the two subtrees connected to *v* with that of the subtree connected to the sibling of *v*. Given an initial gene tree *G* and an edge of *G*, two “alternative” trees can be obtained from *G* by performing an NNI operation (see Figure 4). The hill-climbing proceeds as follows: (1) select a weak edge of *G*; (2) compute the reconciliation cost for the two alternative gene trees obtained by NNI on that edge; (3) if none of these trees decreases the reconciliation cost, then try another weak edge; if none of the weak edges allows to progress, then *G* is a local minimum and the hill climbing stops; (4) otherwise one of the alternative gene trees leads to a decrease in reconciliation cost, and the above process continues with the alternative tree of minimum reconciliation cost. MowgliNNI outputs the final binary rearrangement along with its most parsimonious reconciliation. In the worst cases, MowgliNNI examines all unreliable edges and does not find any better binary rearrangement of the given gene tree *G* since the topology *G* is already (locally) optimal. The whole scheme of *MowgliNNI* is described in Figure 5.

Consider now the time complexity of *MowgliNNI*. Identifying the weak edges is done in *O*(|*G*|) and generating the two alternative gene trees for a NNI operation is done in constant time. Hence, the complexity bottleneck of *MowgliNNI* is the number of times (denoted *N*) the *Θ*(|*S*|^2^ · |*G*|)*Mowgli* algorithm is called. Overall, the time complexity of *MowgliNNI* is *Θ*(|*S*|^2^ · |*G*| · *N*). The next section describes how we can avoid recomputing large parts of the cost matrix, and hence greatly reduce the running time of *MowgliNNI*.

#### ***Combinatorial optimization***

We now present results that take advantage of the way the dynamic programming matrix is computed (Definition 3) to avoid recomputing from scratch the cost matrix associated to a gene tree *G*^′^ obtained by an NNI edit operation from a gene tree *G*. Consider the gene tree *G* of Figure 4, the NNI operation applied on edge (*w*,*v*) that swaps the two subtrees *G*_*b*_ and *G*_*c*_, and the resulting gene tree denoted *G*^′^. We can observe that despite the global architecture of *G* and *G*^′^ differs, the local architectures of subtrees $G_{b},G_{c},G_{d},G_{a_{0}}$, $\ldots G_{a_{k}}$ remain unchanged. Hence, any cost that differs between the matrices *C*(*G*,*S*^′^) and *C*(*G*^′^,*S*^′^) (see Definition 3) is located in a column (i.e. node of the gene tree) associated to an ancestor of *v* (including *v* itself). For each of those nodes, there are two cases: (i) the node belongs to the NNI edge and its two children have subtree that have been modified (e.g. nodes *w* and *v*); (ii) the node is a strict ancestor of the NNI edge (*w*,*v*) and has exactly one child with a subtree that has been modified (e.g. *g*_*k*_,…,*g*_0_).

Lemma 2 below indicates which columns of the cost matrix don’t need to be recomputed.

##### 

**Lemma 2.** Consider a gene tree *G*, the subdivision *S*^′^ of a species tree *S*, an edge (*w*,*v*) of *G*, and the gene tree *G*^′^ obtained from *G* by an NNI operation on (*w*,*v*). For each node *z* of *G* that is not ancestor of *v* in *G* and for each vertex *x* of *S*^′^, then *c*(*z*,*x*) = *c*^′^(*z*,*x*) holds.

This observation results from the fact that the dynamic algorithm of *Mowgli* computes the value of a cell (*z*,*x*) in the cost matrix using cells storing values either for the same node *z* or for its children (see formulas of Definition 3). Hence the value of a cell (*z*,*x*) directly or indirectly depends only on values for cells corresponding to *z* and its descendants. Going from gene tree *G* to *G*^′^ by an NNI operation, precisely changes the descendant relationships of *v* and its ancestors, *i.e.* all other nodes *z* have the same descendants in both *G* and *G*^′^ (see Figure 4), hence *c*(*z*,*x*) = *c*^′^(*z*,*x*) holds for all these nodes.

Unfortunately, there is no extension of Lemma 2 to ensure that when an edge has already been unsuccessfully tried for an NNI, it is useless to reconsider it later, even if it is a descendant in *G* of the edge leading to the last successful NNI.

##### 

**Theorem 1.** Consider a gene tree *G*, the subdivision *S*^′^ of a species tree *S*, an edge (*w*,*v*) of *G*, a gene tree *G*^′^ obtained by an NNI operation on (*w*,*v*), and any strict ancestor *u* of *w* in *G* where the unique child of *u* that is an ancestor of *w* is *u*_1_  w.l.o.g. (i.e. *w* ≤ *u*_1_ in both *G* and *G*’). If *c*(*u*_1_,*x*) ≤ *c*^′^(*u*_1_,*x*) holds for all *x* ∈ *V*(*S*^′^), then *c*(*u*,*x*) ≤ *c*^′^(*u*,*x*) holds for all *x* ∈ *V*(*S*^′^), and as a corollary *C*(*G*,*S*^′^) ≤ *C*(*G*^′^,*S*^′^).

The proof of Theorem 1 is described in Appendix. This theorem leads to the optimized algorithm of *MowgliNNI*, formally stated in Algorithm 1 as an integrated procedure run after *Mowgli*. The later computes a dynamic programming matrix *c* :*V*(*G*) → *V*(*S*^′^) that *MowgliNNI* then partly recomputes given a rearrangement performed on the gene tree *G*. For each rearrangement, the matrix recomputed by *MowgliNNI*, denoted *c*^′^ :*V*(*G*^′^) → *V*(*S*^′^), is obtained in worst case time *O*(|*S*^′^| · *h*(*G*)), where *h*(*G*) is the height of *G* (i.e. the number of its ancestors)

##### **Algorithm 1 *M*** ***o*** ***w*** ***g*** ***l*** ***i*** ***N*** ***N*** ***I*** **(*G*,** ***c*): seeking a gene tree** ***G***^***′***^** of minimum reconciliation cost, starting from a gene tree** ***G* and the precomputed matrix reconciliation cost**$c:V(G)\times V(S^{\prime })\to R$**, where** ***S***^***′***^** is the subdivided species tree.**

##### 

**Theorem 2.** *MowgliNNI* has worst case running time *O*(|*S*|^2^ · |*G*| + |*S*|^2^ · *h*(*G*)·*N*)

Indeed the steps of Algorithm 1 can be described as follows: initializing the reconciliation matrix for the initial gene tree is done in *O*(|*S*|^2^ · |*G*|) time; then updating the matrix for each of the *N* NNIs now only costs *O*(|*S*^′^|  ·  *h*(*G*)) = *O*(|*S*|^2^·*h*(*G*)).

In *MowgliNNI*’s naïve implementation each rearrangement requires to recompute the cost associated to each and every node of the gene tree. In contrast, in the optimized version, an NNI around edge (*w*,*v*) is examined after updating only those costs associated to ancestral nodes of *w*. This has no impact on the worst case complexity (when the gene tree is a caterpillar *h*(*G*) is in *O*(|*G*|)) but significantly reduces the running times in practice since in most cases the number of nodes in *G* is much larger than their average height. For some random tree models the average height of a node in an *n*-leaf tree is indeed proportional to *log*(*n*) [34].

## Results and discussion

### Experiments on simulated datasets

#### ***Simulated gene trees and evolutionary histories***

A phylogeny of 37 proteobacteria was used as a reference species tree (denoted *S*) [8]. Along this tree, we simulated the evolutionary history (denoted *R*_*True*_) of 1000 gene families (*G*_*True*_), each containing from 10 to 100 genes, according to a birth and death process [35]. Birth events can be one of three kinds of evolutionary events, i.e. speciation, duplication, and horizontal gene transfer. During the simulation process along the species tree, a speciation occurs every time a gene lineage reaches an internal node of the species tree, leading to a split in two gene lineages. A birth event happening strictly between two nodes of the species tree can only correspond to a gene duplication or a horizontal gene transfer event. A birth is decided to be duplication or a transfer according to the input rates of these events.

The death of a gene lineage corresponds to a loss event, which happens according to an input loss rate. The species tree was scaled to the height of 500 million years (Mya). The speciation rate is determined by the topology and the height of the species tree. Each of the 1000 gene families was generated with different event rates, the loss rate being randomly chosen in the range [0.0010–0.0018] events/gene per million year. The ratio between the sum of duplication and transfer rates and the loss rate was randomly chosen in the range [0.5 - 1.1], the duplication rate being [70% - 100%] of the mentionned sum. This birth and death process first output a complete gene tree *G*^*o*^, then the “true” gene tree *G*_*True*_ was obtained from *G*^*o*^ by pruning extinct subtrees. The “true” evolutionary events to be recovered by the reconciliation programs are those appearing in *G*_*True*_. We denote *R*_*True*_ the history composed by these events. We only considered gene families containing at most ten duplication and transfer events in their true evolution. In particular for the transfer events, this constraint allowed us to limit the number of cases where the true evolution contains a sequence of consecutive transfers where non-transferred genes are lost (i.e. a sequence ofTL events). Such a piece of history can hardly be recovered by reconciliation methods as it left no trace at all in the gene tree.

**Figure 6:**
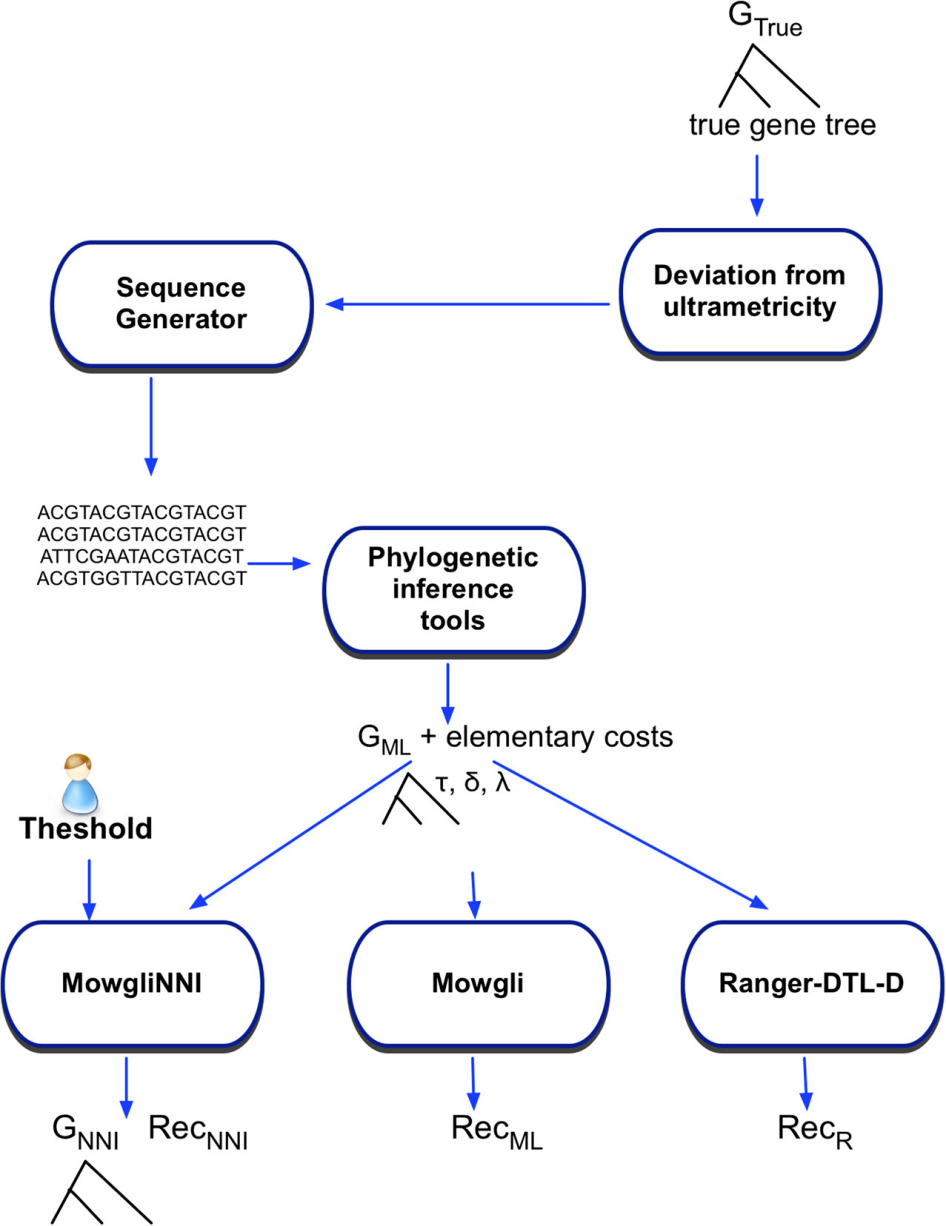
**The simulation protocol to obtain inferred gene trees from sequences derived from a true gene tree.** The module “*Deviation from ultrametricity*” is taken from the program of Galtier that converts the edge lengths of ultrametric trees from the time unit into substitution numbers [36]. *G*_*ML*_ denotes the initial gene tree inferred by Maximum Likelihood from the simulated sequences; *Rec*_*ML*_, resp. *Rec*_*R*_, denotes the reconciliation between *G*_*ML*_ and the reference species tree predicted by *Mowgli*[2], resp. Ranger-DTL-D [3]; *G*_*NNI*_ is the alternative gene tree found by *MowgliNNI* and *Rec*_*NNI*_ is the reconciliation between *G*_*NNI*_ and the species tree.

Starting from *G*_*True*_, a further step of the simulation protocol allows to obtain estimates of both this gene tree and the events composing its history (see Figure 6).

The length of the edges in *G*_*True*_ were first converted from duration to number of substitutions per site by a module taken from [36]. Next, we simulated the evolution of 1500 - 3000 bp DNA sequences along this tree under the GTR model, thanks to the Seq-Gen program [37]. The alignment, composed of one sequence per extant gene, was then given as input to RAxML [38] to obtain a maximum likelihood estimate of the gene tree, denoted *G*_*ML*_ (also called *initial* gene tree below). *Mowgli*[2] and Ranger-DTL-D [3] were then used to infer a most parsimonious evolutionary history *R*_*ML*_, resp. *R*_*R*_, between this initial gene tree and the reference species tree *S*. Separately, *MowgliNNI* was used to search for an alternative gene tree topology (*G*_*NNI*_) of lower reconciliation cost, along with one of its most parsimonious evolutionary history (*R*_*NNI*_). The elementary cost considered for each event kind (with being**,** or) was computed as follows:

(1) $$\text{Cos}t_{E}=\left\{\begin{array}{ll} \text{log}\left(\right)\frac{D_{R_{\text{True}}}+T_{R_{\text{True}}}+L_{R_{\text{True}}}}{E_{R_{\text{True}}}})) & \text{if}E_{R_{\text{True}}}\neq 0\\ \text{log}\left(\right)\frac{D_{R_{\text{True}}}+T_{R_{\text{True}}}+L_{R_{\text{True}}}}{0.1})) & \text{otherwise} \end{array}\right)$$

where$E_{R_{\text{True}}}$ stands for the number of events of the corresponding kind in *R*_*True*_.

#### ***Measuring the accuracy***

First, we estimated the improvement in the accuracy of the gene tree’s topology, as measured by the Robinson-Foulds (RF) distance [39] between the true gene tree (*G*_*True*_) and the inferred gene tree (*G*_*ML*_). As a second measurement of the accuracy of inferred reconciliations we compared the positions of, and events predicted by *MowgliNNI* and *Mowgli* with those present in the true history. This is achieved by studying the proportion of true positive (TP), false positive (FP) and false negative (FN) separately for duplications, transfers and losses [2]. True negatives (TN) were not studied as their number is considerably large (if even finite) and hard to determine. An event of *R*_*True*_ is declared as correctly predicted when it concerns the right part of the gene tree (node or edge) placed in the correct branch or node of the species tree (see [2] for more details). Incidentally, both the receiver and the donor edge of the species tree have to be correctly indicated for a predicted transfer event to be declared as correct.

#### ***MowgliNNI provides more accurate inferences***

We explored the ability of *MowgliNNI* to improve the set of *G*_*ML*_ trees using six different bootstrap values as threshold for defining weak edges, i.e. 20, 40, 60, 80, 90, and 95. The *G*_*ML*_ trees were inferred from relatively long sequences, they thus contained a large proportion of high bootstrap values, *e.g.* more than 63% edges had a bootstrap value ≥ 80. Though this left only a moderate number of edges in each gene tree to be considered by *MowgliNNI* for rearrangement, the method was still able to improve their quality (see below).

**Figure 7:**
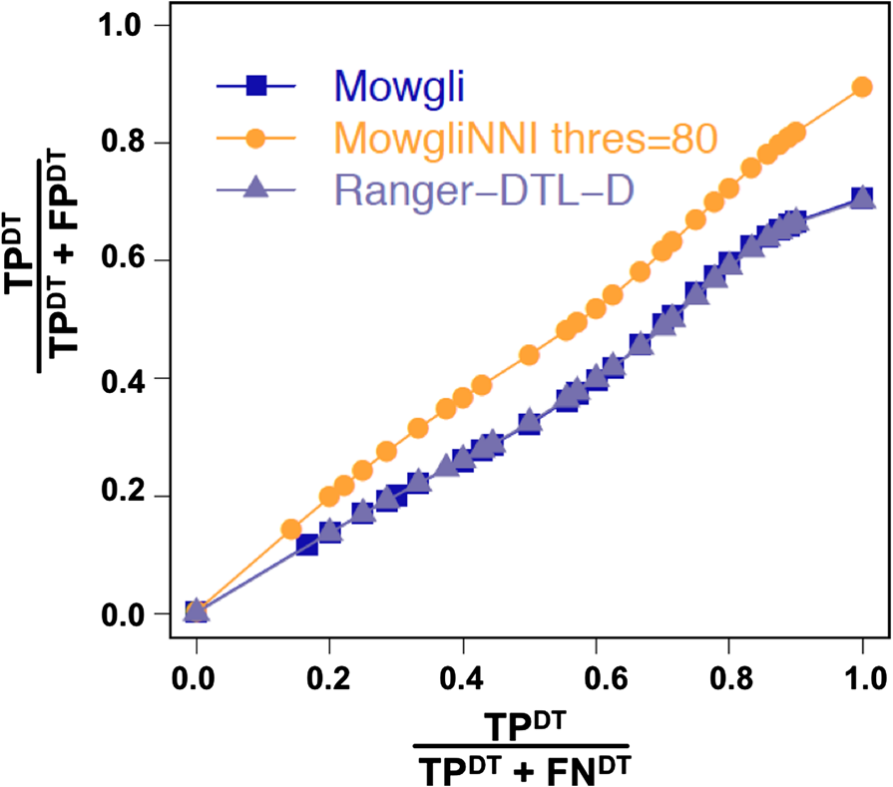
**Impact of *MowgliNNI* on the proportion of True Positive events.** The accuracy of *Mowgli*, Ranger-DTL-D and *MowgliNNI* (threshold=80) in inferring duplications and transfers, where ***TP***^***DT***^ (resp. ***FP***^***DT***^, ***FN***^***DT***^) denotes the true positive (resp. false positive, false negative) of duplications and transfers predicted.

*Mowgli* and Ranger-DTL-D showed a similar accuracy in inferring duplications and transfers (Figure 7), though Ranger-DTL-D proposed reconciliations with higher costs in 13% of the cases. As both methods reconciled the same trees and used the same elementary costs for the events, one might wonder why they did not always obtain the same reconciliation costs. This results from different factors among which the most important is that Ranger-DTL-D relies on a less general reconciliation model than *Mowgli* (e.g. not ensuring time consistency and not allowing gene loss in the donor branch after a transfer), but which on the other hand allows it to run at greater speed. As *Mowgli* and Ranger-DTL-D performed similarly, in the following we just report results obtained with *Mowgli*.

*MowgliNNI* progressively reduced the number of predicted duplications, transfers and losses as the threshold increased. At threshold 0 (where *MowgliNNI* = *Mowgli*, 5510 duplications, 2494 transfers and 12190 losses were predicted on the whole dataset; going to threshold 80, these numbers dropped to 4602 duplications, 1676 transfers and 8133 losses, *i.e.* values that are much closer to the 4535 duplications and 8260 losses contained in the true reconciliations.

**Figure 8:**
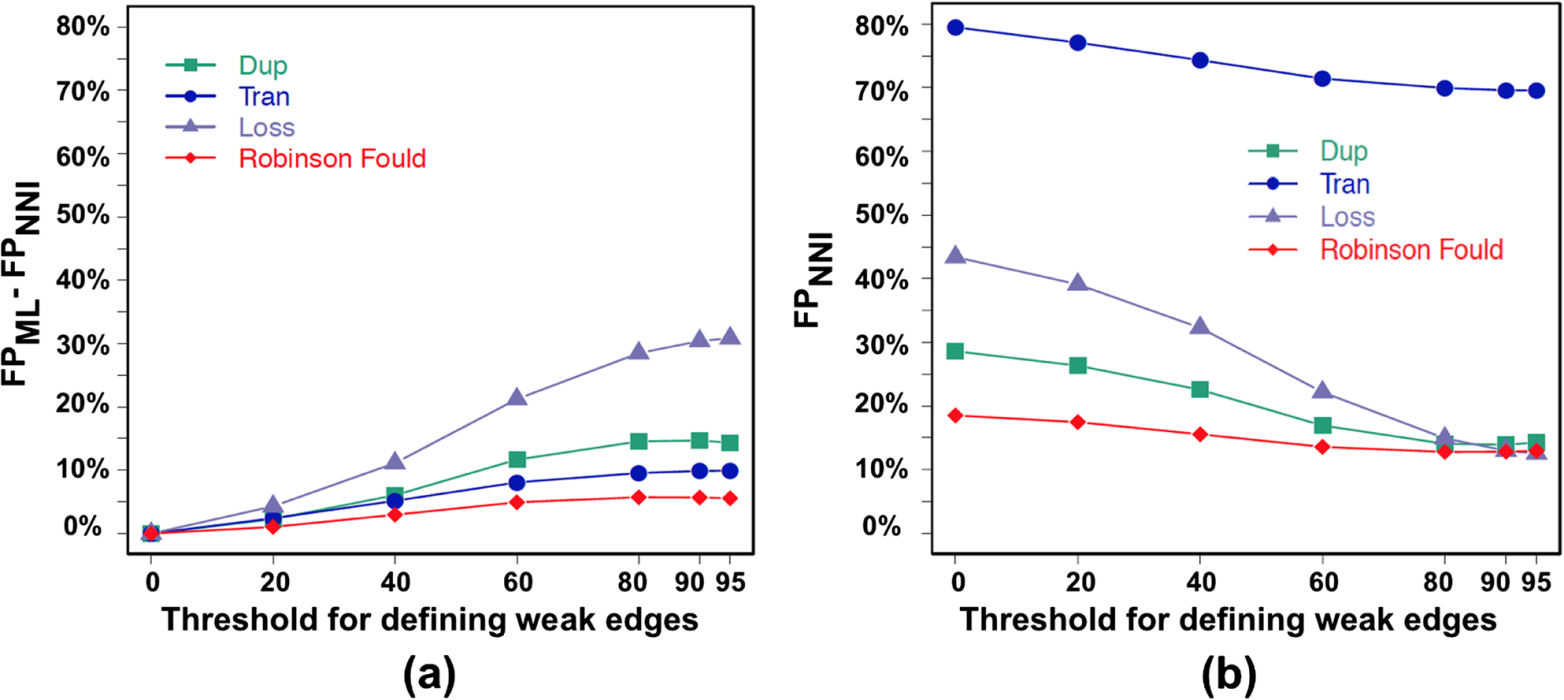
**Impact of *MowgliNNI* on the proportion of False Positive events.(a)** Average false positive error reduction achieved by NNI trees (***FP***_***NNI***_) w.r.t. that of the initial gene trees (***FP***_***ML***_). The positive value indicate that the NNI tree has on average less errors than the initial tree. **(b)** Average FP of the NNI trees – note that values at threshold 0 correspond to the FP of *Mowgli*.

Figure 8(a) shows that, no matter the threshold value, the false positive (FP) of *MowgliNNI* are always less than that of *Mowgli* both in terms of RF distance and evolutionary events (duplications, transfers and losses). This means that the *G*_*NNI*_ trees are closer to the true ones than the initial *G*_*ML*_ trees inferred from sequences only. Similarly, the evolutionary events inferred from the *G*_*NNI*_ trees are more accurate. As the threshold increases from 0 to 80, *R*_*NNI*_ contains less and less FP events, hence widening the gap in accuracy between *Mowgli* and *MowgliNNI*. As increasing the threshold results in reconsidering a larger number of *G*_*ML*_ edges for NNI operations, this means that the species tree examined through the reconciliation lens is a good guide tree for correcting wrong edges of the gene trees.

**Figure 9:**
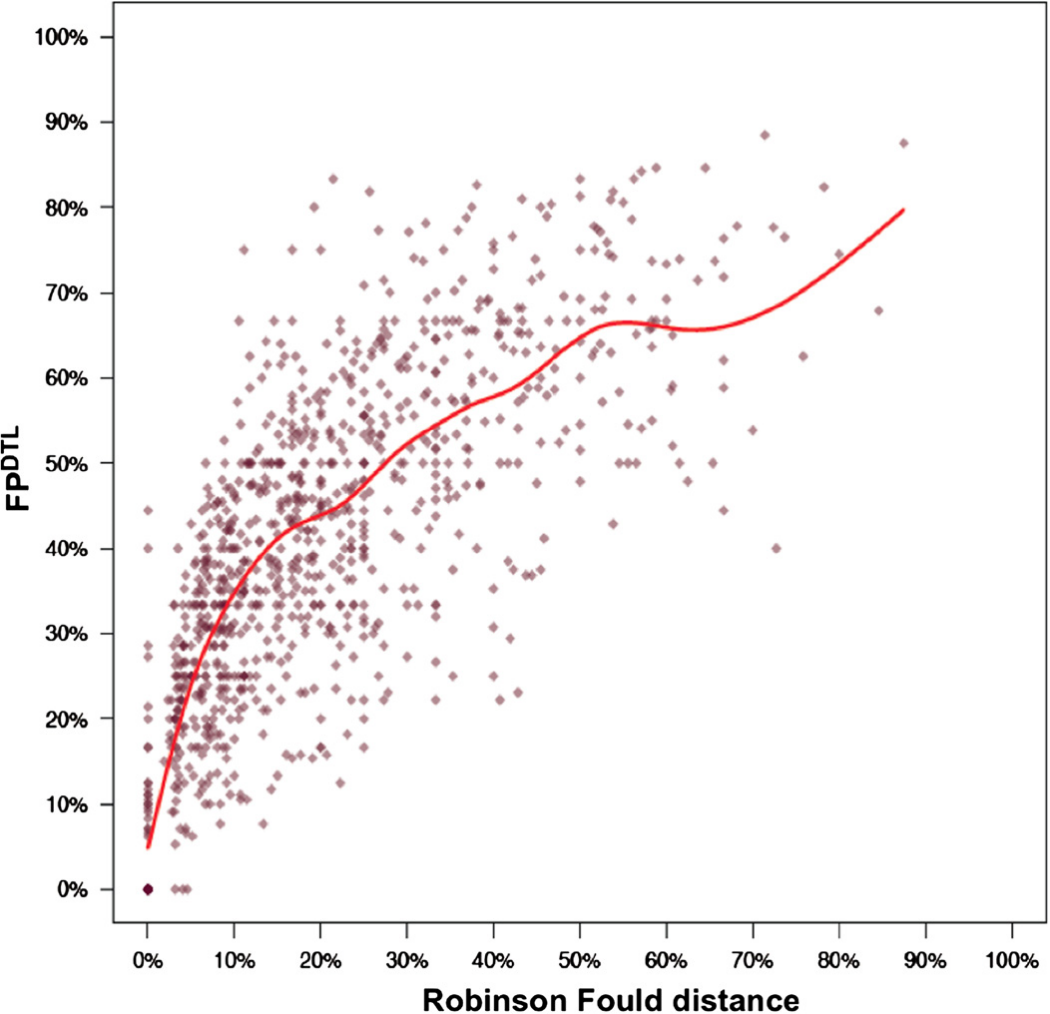
**Impact of gene tree errors on reconciliation accuracy.** The false positive (***FP***^***DTL***^) error rate of the events predicted by reconciliation methods grows exponentially with respect to the Robinson Foulds distance between the initial and true tree.

**Figure 10:**
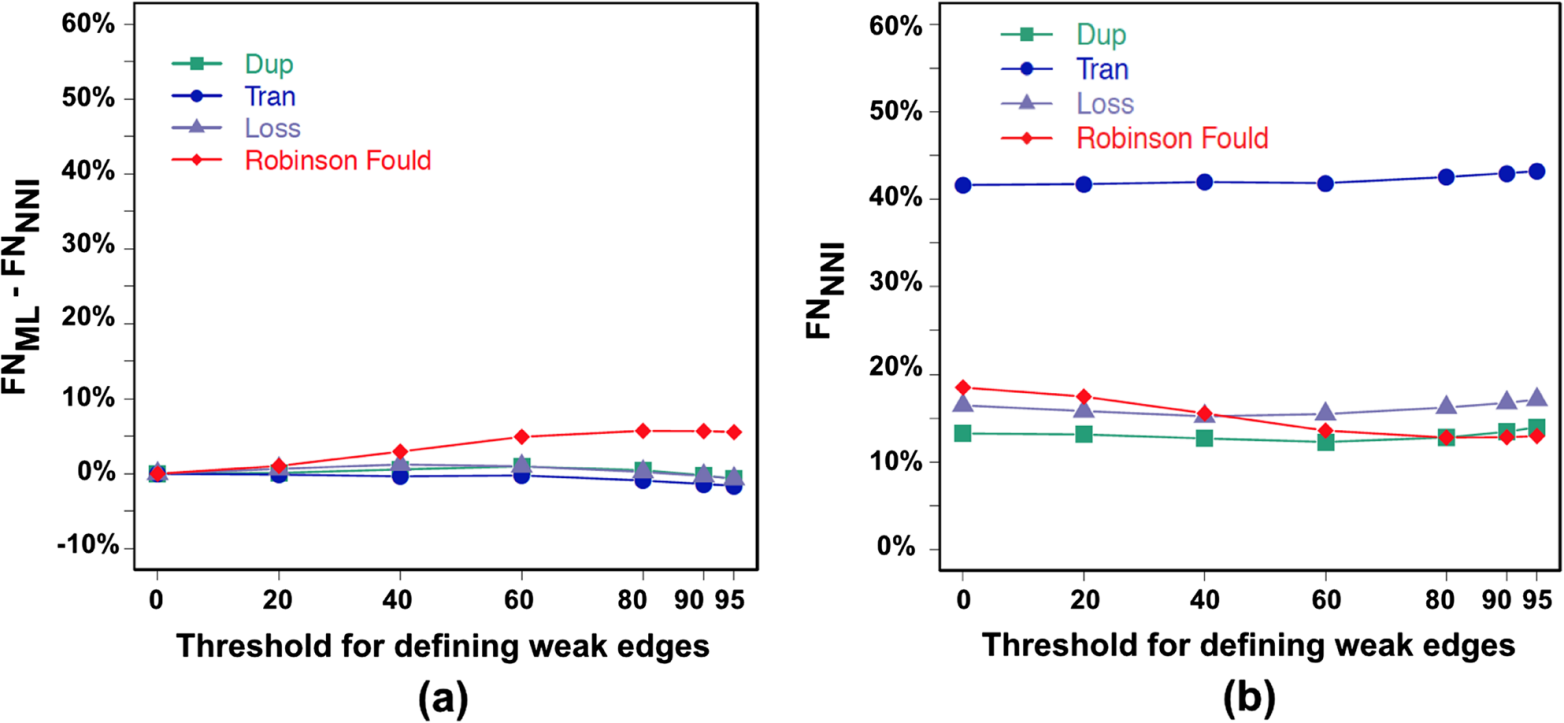
**Impact of *MowgliNNI *on the proportion of False Negative events.(a)** The false negative reduction of NNI gene trees (***FN***_***NNI***_) in comparison to the initial gene trees (***FN***_***ML***_). While reducing the number of wrong events predicted, *MowgliNNI* mostly does not remove the events that have been correctly predicted. **(b)** FN of the NNI trees. FN and FP of Robinson Foulds distance are the same since the true, initial and NNI gene trees are binary.

The average number of false positive events of the *R*_*NNI*_ reconciliations decreases as the threshold increases (Figure 8(b)). However, as in Doyon et al. [2], the average number of FP transfers is quite high compared to that of duplications and losses. This can be explained by several reasons. First, a transfer is judged incorrect as soon as *(i)* it does not depart or end in the same edges of the species tree as the corresponding true transfer, or *(ii)* it does not concern the same edge in the gene tree. Overall, there is an additional constraint w.r.t. duplications and loss events, leading on average to more incorrect events. This point is all the more sensitive that several most parsimonious reconciliations (MPR) are obtained in a number of cases, while we just accounted for one of them for each gene family. Hence, event error rates we report are pessimistic, and especially for transfers due to the stringent conditions for judging a transfer as correct. Note that the multiplicity of MPRs does not affect the RF error terms. Last, incorrect gene trees lead to incorrect event inferences, but the latter are very sensitive to only small errors in gene trees. The event FP error grows almost exponentially when the RF distance between the initial and the true tree increases from 0 to 10% (Figure 9). Figures 8 and 10 show that transfers are more influenced by this factor, as a result of more stringent conditions for being correct.

#### ***Influence of the sequence length parameter***

*MowgliNNI* achieved a higher improvement over *Mowgli* on the subset of 327 *G*_*ML*_ families inferred from *short* sequences (length 1500–2000 bp) than on the subset of 336 families inferred from *long* sequences (length 2500–3000 bp), see Table 1. For instance, at threshold 80, *MowgliNNI* was able to proposed a modified gene tree (*G*_*NNI*_) for up to 83%, resp. 92%, of the families containing weak edges when *G*_*ML*_ was inferred from long, resp. short, sequences. Similar results were observed for the quality of modified gene trees(*G*_*NNI*_) in term of RF distance to *G*_*True*_ and for the quality of reconciliations in term of event distance between inferred and true reconciliation. The fact that higher improvements are obtained for shorter sequences was confirmed through the simulation of 1000 *G*_*ML*_ families inferred from much shorter sequences (400 bp), where still a higher improvements where obtained (see Table 2).

**Table 1:** **Quality of the gene trees (*G***_***NNI***_**) and reconciliations (*R***_***NNI***_**) inferred by *MowgliNNI * depending on the length of the sequences used to obtain** ***G***_***ML ***_**trees and on the threshold indicating weak edges**

	**Short sequences**			**Long sequences**		
**Threshold**	**20**	**80**	**95**	**20**	**80**	**95**
**Number of gene families containing weak edges**	163	323	327	118	328	332
**%cases s.t. *C o s t *** **( *S *,** ***G***_***N *** ***N *** ***I***_**) <** ***C o s t *** **( *S *,** ***G***_***M*** ***L***_**)**	80	92	91	75	83	84
**%cases s.t. *R F *** **(*G***_***T *** ***r *** ***u *** ***e***_**,** ***G***_***N *** ***N *** ***I***_**) <** ***R F *** **(*G***_***T *** ***r *** ***u *** ***e***_**,** ***G***_***M*** ***L***_**)**	43	74	73	29	67	67
**%cases s.t. *R F *** **(*G***_***T *** ***r *** ***u *** ***e***_**,** ***G***_***N *** ***N *** ***I***_**) = *R F *** **(*G***_***T *** ***r *** ***u *** ***e***_**,** ***G***_***M*** ***L***_**)**	53	17	18	67	26	24
**%cases s.t. *R F *** **(*G***_***T *** ***r *** ***u *** ***e***_**,** ***G***_***N *** ***N *** ***I***_**) >** ***R F *** **(*G***_***T *** ***r *** ***u *** ***e***_**,** ***G***_***M*** ***L***_**)**	4	9	9	4	7	9
**%cases s.t. *E D *** **(*R***_***T *** ***r *** ***u *** ***e***_**,** ***R***_***N *** ***N *** ***I***_**) <** ***E D *** **(*R***_***T *** ***r *** ***u *** ***e***_**,** ***R***_***M*** ***L***_**)**	66	82	83	51	76	76
**%cases s.t. *E D *** **(*R***_***T *** ***r *** ***u *** ***e***_**,** ***R***_***N *** ***N *** ***I***_**) = *E D *** **(*R***_***T *** ***r *** ***u *** ***e***_**,** ***R***_***M*** ***L***_**)**	24	12	12	33	20	19
**%cases s.t. *E D *** **(*R***_***T *** ***r *** ***u *** ***e***_**,** ***R***_***N *** ***N *** ***I***_**) >** ***E D *** **(*R***_***T *** ***r *** ***u *** ***e***_**,** ***R***_***M*** ***L***_**)**	10	6	5	16	4	5

Threshold of 20, 80 and 95 bootstrap support values are reported and sequence alignments of length in the range 1500 - 2000 bp (*short seq.*) or 2500–3000 bp (*long seq.*) were considered. For each threshold, the second row indicates the number of gene families (*G*_*ML*_) containing weak edges among the 327 (resp. 336) families inferred from short (resp. long) sequences. The third row indicates the percentage of these families where *MowgliNNI* proposes a modified tree of lower reconciliation cost. The last six rows provide the percentage of the former families where *MowgliNNI* provides modified gene trees (resp. reconciliations) that are closer, equally far or farther from the true gene trees (resp. the true reconciliations). *RF*(*G*_*True*_,*G*_*X*_) denotes the Robinson Foulds distance between *G*_*True*_ and *G*_*X*_, *ED*(*R*_*True*_,*R*_*X*_) = |*R*_*True*_ - *R*_*X*_| + |*R*_*X*_ - *R*_*True*_|, where *X* stands for NNI or ML.

**Table 2:** **Quality of the gene trees (*G***_***NNI***_**) and reconciliations (*R***_***NNI***_**) inferred by *MowgliNNI * on very short sequences**

**Threshold**	**20**	**80**	**95**
**Number of gene families containing weak edges**	794	1000	1000
**%cases s.t. *C o s t *** **( *S *,** ***G***_***N *** ***N *** ***I***_**) <** ***C o s t *** **( *S *,** ***G***_***M*** ***L***_**)**	89	97	97
**%cases s.t. *R F *** **(*G***_***T *** ***r *** ***u *** ***e***_**,** ***G***_***N *** ***N *** ***I***_**) <** ***R F *** **(*G***_***T *** ***r *** ***u *** ***e***_**,** ***G***_***M*** ***L***_**)**	58	77	75
**%cases s.t. *R F *** **(*G***_***T *** ***r *** ***u *** ***e***_**,** ***G***_***N *** ***N *** ***I***_**) = *R F *** **(*G***_***T *** ***r *** ***u *** ***e***_**,** ***G***_***M*** ***L***_**)**	39	16	16
**%cases s.t. *R F *** **(*G***_***T *** ***r *** ***u *** ***e***_**,** ***G***_***N *** ***N *** ***I***_**) >** ***R F *** **(*G***_***T *** ***r *** ***u *** ***e***_**,** ***G***_***M*** ***L***_**)**	3	7	9
**%cases s.t. *E D *** **(*R***_***T *** ***r *** ***u *** ***e***_**,** ***R***_***N *** ***N *** ***I***_**) <** ***E D *** **(*R***_***T *** ***r *** ***u *** ***e***_**,** ***R***_***M*** ***L***_**)**	78	91	91
**%cases s.t. *E D *** **(*R***_***T *** ***r *** ***u *** ***e***_**,** ***R***_***N *** ***N *** ***I***_**) = *E D *** **(*R***_***T *** ***r *** ***u *** ***e***_**,** ***R***_***M*** ***L***_**)**	15	5	5
**%cases s.t. *E D *** **(*R***_***T *** ***r *** ***u *** ***e***_**,** ***R***_***N *** ***N *** ***I***_**) >** ***E D *** **(*R***_***T *** ***r *** ***u *** ***e***_**,** ***R***_***M*** ***L***_**)**	7	4	4

For each tested threshold value, the second row indicates the number of gene families (*G*_*ML*_) containing some weak edges among 1000 families inferred from very short sequences of length 400 bp. Third row indicates the percentage of these families where *MowgliNNI* proposes a modified tree of lower reconciliation cost. The last six rows provide the percentage of the former families where *MowgliNNI* provides modified gene trees (resp. reconciliations) that are closer, equally far or farther from the true gene trees (resp. the true evolutions). *RF*(*G*_*True*_,*G*_*X*_) denotes the Robinson Foulds distance between *G*_*True*_ and *G*_*X*_, *ED*(*R*_*True*_,*R*_*X*_) = |*R*_*True*_ - *R*_*X*_| + |*R*_*X*_ - *R*_*True*_|, where X stands for NNI or ML.

#### ***Robustness of reconciliations to imprecision in the event costs***

In order to measure the dependance of *MowgliNNI* on the precise costs used for each kind of event, we ran the method with the threshold *t* = 80 on *G*_*ML*_ trees with costs varying up to 10%, 20%, then 50% w.r.t. those used derived from *R*_*True*_ using Formula (1). The paired *t*-test for RF distances shows that the *G*_*NNI*_ trees obtained with the new noisy costs are not significantly different from those obtained with the former costs (p-value=0.1747, 0.1758, 0.1144 respectively). The accuracy of inferred events also does not change much. Transfers have the highest variation with 4.2% (resp. 3.1%) increase in FP (resp. FN) when the event costs vary up to 50% (Table  3). Thus, *MowgliNNI* is quite robust to changes in the event costs.

**Table 3:** **The robustness of *MowgliNNI * to changes in event costs with respect to the initial ones computed by Formula (**1**) (Column 1)**

**Event cost variation**	**RF**	**FP Dup**	**FP Tran**	**FP Loss**	**FN Dup**	**FN Tran**	**FN Loss**
0%	12.8	14.1	69.9	14.9	12.8	42.5	16.2
10%	12.9	14.3	72.1	16.4	13.0	45.0	17.6
20%	12.9	14.4	71.1	16.8	13.2	44.2	17.7
50%	12.9	14.6	74.1	17.8	15.0	45.6	18.9

Error terms (in %) are the Robinson Foulds distances (RF) as well as the false positive (FP), and false negative (FN) for the various event predicted. Non-negligible variations in event costs result in small variations of the error terms (transfers being the most affected).

#### ***Room for future improvement***

To measure how much of the achievable improvement over the *G*_*ML*_ trees was realized by *MowgliNNI*, we studied the distribution of reconciliation costs of all possible gene trees for several cases involving a computationally manageable number of species. The shape of the distribution together with the relative position of the costs obtained for *G*_*True*_, *G*_*ML*_ and *G*_*NNI*_ within those distributions gives information on how much improvement could be achieved in the future by more sophisticated methods (e.g., relying on SPR moves). We report here on two cases representative of our observations: two true gene trees$G_{\text{True}}^{A},G_{\text{True}}^{B}$ of 8 taxa were generated from the species tree *S* of 37 proteobacteria according to the protocol described in Figure 6. Their two associated histories *A* and *B* were used as starting points to obtain both sequence alignments and reconciliations costs (according to Equation 1). This time, 50 sequence alignments were generated from each of the two gene trees. A maximum likelihood tree was obtained from each of the 100 alignments, with bootstrap supports associated to its edges. These trees were then submitted for improvement to *MowgliNNI*, applying a threshold 50 to specify weak edges, and relying on event costs corresponding to histories A and B respectively. All reconciliations were performed with respect to the species tree *S*.

**Figure 11:**
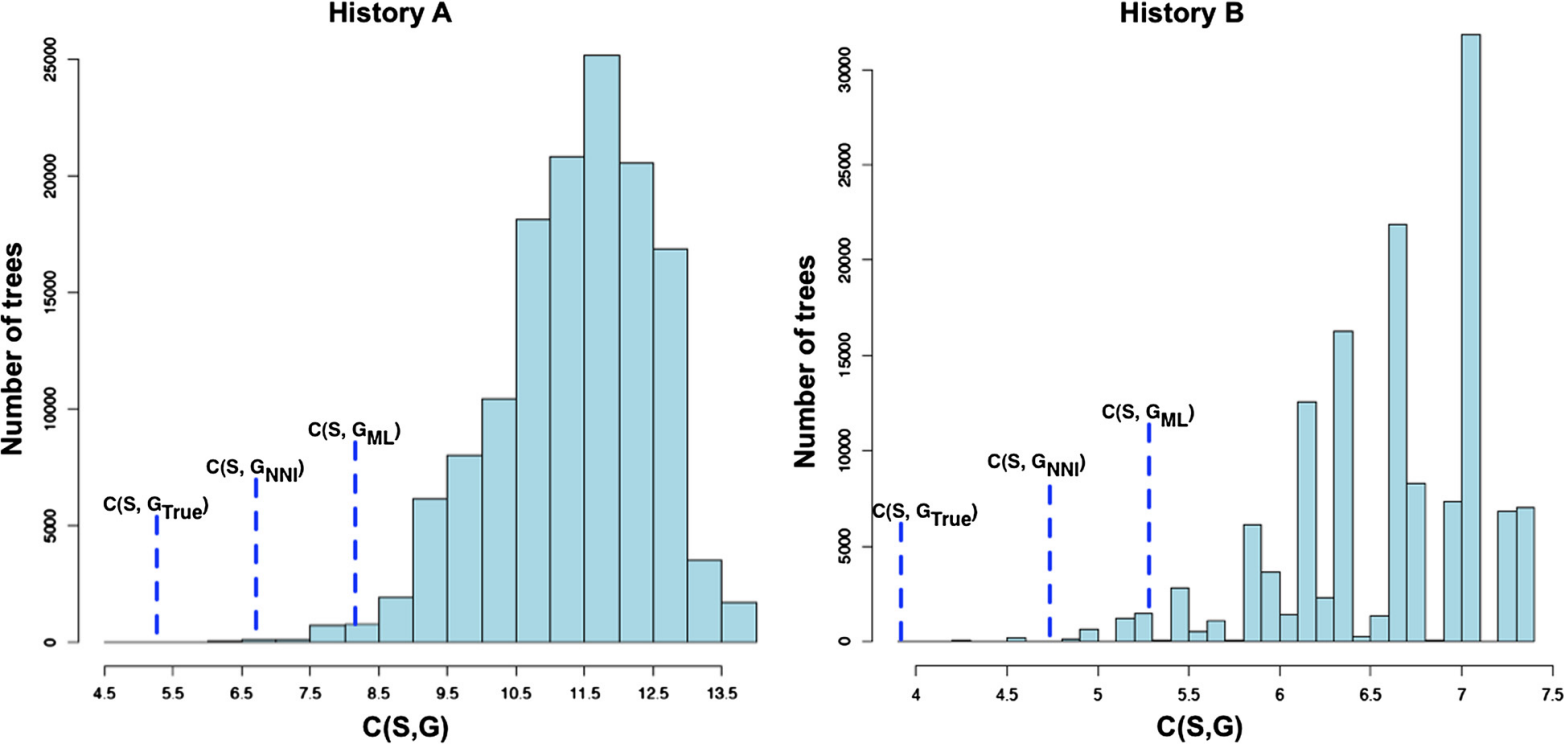
**Distributions of reconciliation costs*C(S,G) *over all possible binary gene trees*G *for two sets of 8 taxa from the phylogeny*S *of 37 proteobacteria, obtained by generating two simulated histories A and B along*S*.** For each distribution, we indicate the position of *C*(*G*_*True*_,*S*), the reconciliation cost obtained by *Mowgli* for the true gene tree of the corresponding history. The plot also indicates the average cost *C*(*S*,*G*_*ML*_) obtained for reconciliations from maximum likelihood trees and the average cost *C*(*S*,*G*_*NNI*_) obtained for reconciliations of *MowgliNNI* trees obtained from the maximum likelihood trees.

Figure 11 shows the distributions of reconciliation costs *C*(*S*,*G*) obtained for all possible binary trees *G* having the same leaves as$G_{\text{True}}^{A}$** and**$G_{\text{True}}^{B}$ respectively. The first observation is that though the same species tree was used in both cases, these distributions vary significantly in range and shape depending on the gene tree leaf set. In the case of history B, the number of trees with reconciliation costs falling in a given range varies sporadically, whereas the number of trees in a given range almost follows a normal (or beta) distribution for history A.

History A involved 2 duplications, no transfer and 7 losses and was correctly recovered by the parsimonious reconciliation of *Mowgli* from$G_{\text{True}}^{A}$. However, History B (involving 2 duplications, 1 transfer and 5 losses) was incorrectly recovered from$G_{\text{True}}^{B}$, the achieved$C(G_{\text{True}}^{B},S)=3.98$ cost being less than the 5.75 cost for the real history. Though the real cost is in the left part of the distribution, it is not the minimum point of the distribution, showing that parsimony can sometimes be misleading when followed to its extreme.

Nevertheless, in both cases, the true gene tree is among the ones having the minimum reconciliation costs: it is precisely the one leading to the minimum cost for history A and among the nine best trees for history B. On these examples (and other cases not shown), parsimony can be considered as a very good guide towards the correct gene tree, even if the reconciliation from this correct tree can underestimate the number of real events (as discussed above).

For both histories A and B, *MowgliNNI* proposed a gene tree *G*_*NNI*_ whose reconciliation cost was on average closer from that of the true gene tree – and from the real cost – than the cost obtained from the maximum likelihood tree.

#### ***Conclusion on simulated datasets***

In summary, *MowgliNNI* successfully uses the reconciliation cost as additional information to resolve the uncertain parts of gene trees inferred from sequences only. Though the gene tree resolutions are partly guided by reconciliations with the species tree, they are not attracted away from the true gene trees, but are closer to them than the initial gene trees. As a result, *MowgliNNI* infers gene events more accurately, which is of prior importance to distinguish orthologs from paralogs and xenologs [14].

### Experiments on real data

As species tree *S*, we chose a phylogeny covering 336 genomes of Bacteria and Archaea recently inferred by Abby et al. [30].

Then, a dataset of 29,709 homologous gene families spanning these taxa was collected from the HOGENOM database (release 04) [40]. Each such family contains from 3 to 312 taxa. The gene tree of each family from this dataset was reconciled with the species tree by *Mowgli* and *MowgliNNI* using costs *τ* = 3, *δ* = 3.5, resp. *λ* = 1 for transfers, duplications, resp. losses. These costs were estimated on the basis of several bacteria phyla by a maximum likelihood method [18,41]. A threshold of 50% for branch support values was used to indicate to *MowgliNNI* the weak edges in the gene trees.

#### ***A decrease in the number of inferred events and reconciliation costs***

*MowgliNNI* allowed to change the gene tree, hence to lower the reconciliation cost, in 24% of the ≈30,000 families. This gain is non-negligible and has a real importance as changing the gene tree topology has an important impact on the inferred events (as already shown on simulated datasets and discussed below). In turn, these inferred events may serve to predict the function of new sequences on the basis of their orthology relationships with annotated sequences, orthology following from the chosen reconciliation. Among previous reconciliation studies that allowed to modify the gene trees, Berglund-Sonnhammer et al. report that 10% of their families were improved [21] when allowing rearrangements on weak edges under the DL model, while Chaudhary et al. improved all their gene trees in a pure D model when rearranging gene trees with *Subtree Prune and Regraft* (SPR) operations [24]. Note that the heterogeneity of models and datasets used in these studies limit the comparison of their results, but we cite them for completeness.

For gene families with a lower reconciliation cost (24% of all families), we counted the number of events of each kind (D,T,L) inferred by *Mowgli* and *MowgliNNI*. As a rule, *MowgliNNI* led to a decrease in the number of events in inferred evolutionary histories. In particular, the number of transfers is reduced in 88.3% of these gene families, the number of losses being reduced in 59.9%, while the number of duplications is almost the same (decrease in 5.2*%* of the families). These results obtained in the DTL model echo those of Durand et al. reporting that in the DL model gene tree rearrangements substantially reduce the number of events needed to explain the data [19]. The differences in reductions we observed among the kind of events can be explained by the costs – estimated from [18,41] – that we used for the events (*τ* = 3,*δ* = 3.5,*λ* = 1). Given those costs, it is usually more parsimonious to explain the conflicts between a gene and the species tree by a combination of and rather than a combination of, and. Thus, when *MowgliNNI* infers a gene tree closer to the species tree, it mostly removes the need for artificial transfers (and losses to a lesser extent), while not altering that much the number of duplications.

**Figure 12:**
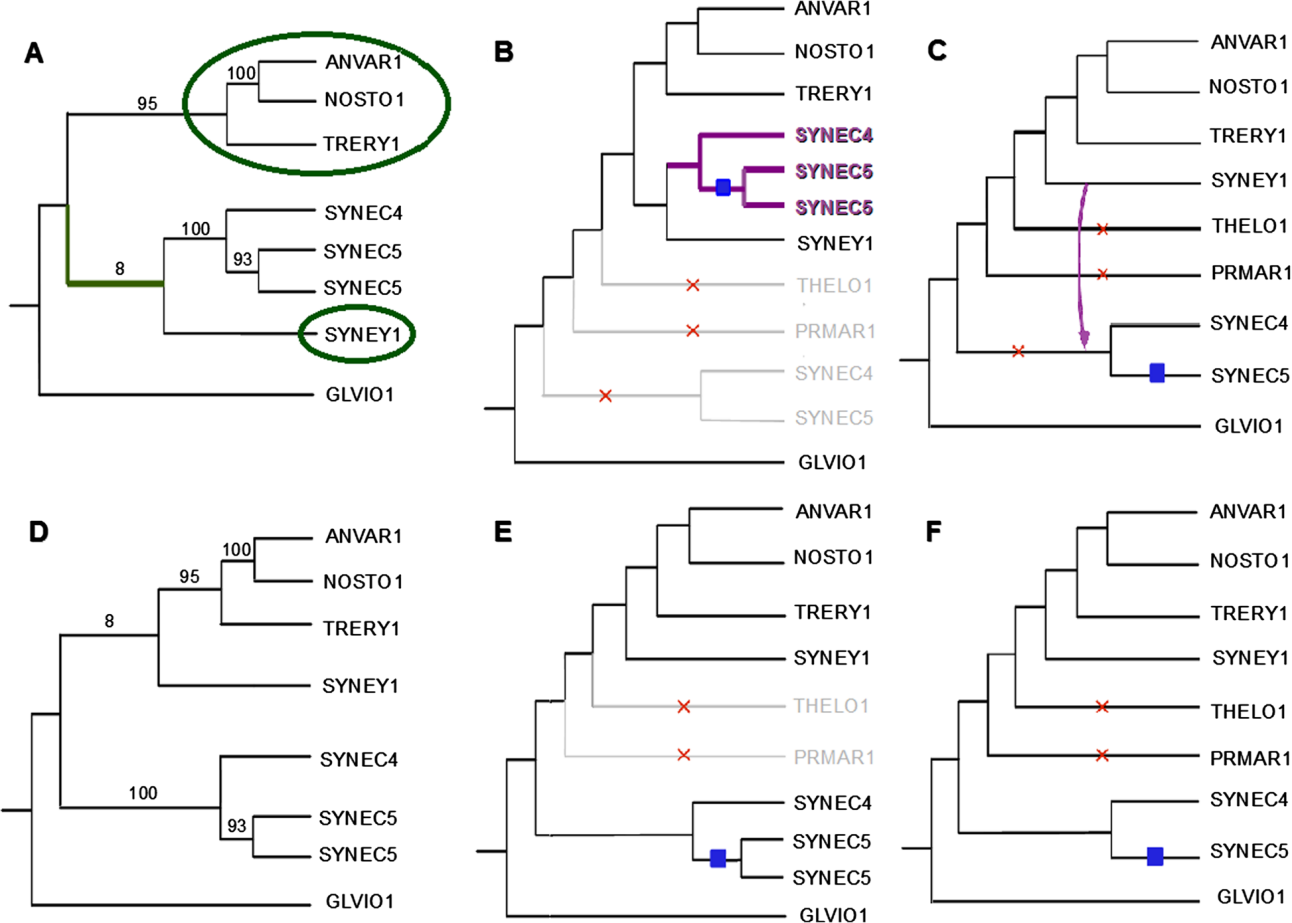
**Example of reconciliation with*Mowgli *(top) and *MowgliNNI *(bottom).A** and **D** are the gene trees. The NNI rearrangement around the green bold edge in **A** exchanging the two subtrees in green circles results in **D**. **B** and **E** are the reconciled gene trees showing the duplications (blue squares), losses (red crosses) and the transferred subtree (purple). **C**, resp. **F**, is the species tree with the events inferred by *Mowgli*, resp. *MowgliNNI*, mapped onto the appropriate edges (a purple arrow shows the origin and destination of the transfer). NOSTO1 - *Nostoc sp. PCC 7120*; ANVAR1 - *Anabaena variabilis ATCC 29413*; TRERY1 - *Trichodesmium erythraeum IMS101*; SYNEC4 - *Synechococcus sp. JA-2–3B’a(2–13)*; SYNEC5 - *Synechococcus sp. JA-3–3Ab*; SYNEY1 - *Synechocystis sp. PCC 6803*; GLVIO1 - *Gloeobacter violaceus PCC 7421*; THELO1 - *Thermosynechococcus elongatus BP-1*; PRMAR1 - *Prochlorococcus marinus str. MIT 9312*.

To give a precise example of how *MowgliNNI* proposes a more parsimonious reconciliation on a modified gene tree than *Mowgli* on the initial gene tree, Figure 12 details the particular case of family HBG040981, (a putative tocopherol cyclase). In this case, *MowgliNNI* proposes a gene history resorting to less events by rearranging an edge with a very small support. The gene tree modified by *MowgliNNI* leads to a reconciliation having one transfer and one loss less than the reconciliation performed from the initial gene tree. On the whole, the reconciliation cost goes from 9.5 down to 5.5.

#### ***A decrease in the number of equally most parsimonious reconciliations***

In addition to reductions in number of events and hence reconciliation cost, the modified gene trees proposed by *MowgliNNI* usually reduced the number of alternative MPRs, i.e. equally most parsimonious histories. On a random sample of two dozens modified gene trees, the number of MPRs is reduced in 63% of the cases (by a factor of 18 in the best case), and increased in 21% (by a factor 3 at worst). This echoes similar observations done by other authors.

#### ***The improvement in running time due to the optimized version of MowgliNNI***

**Figure 13:**
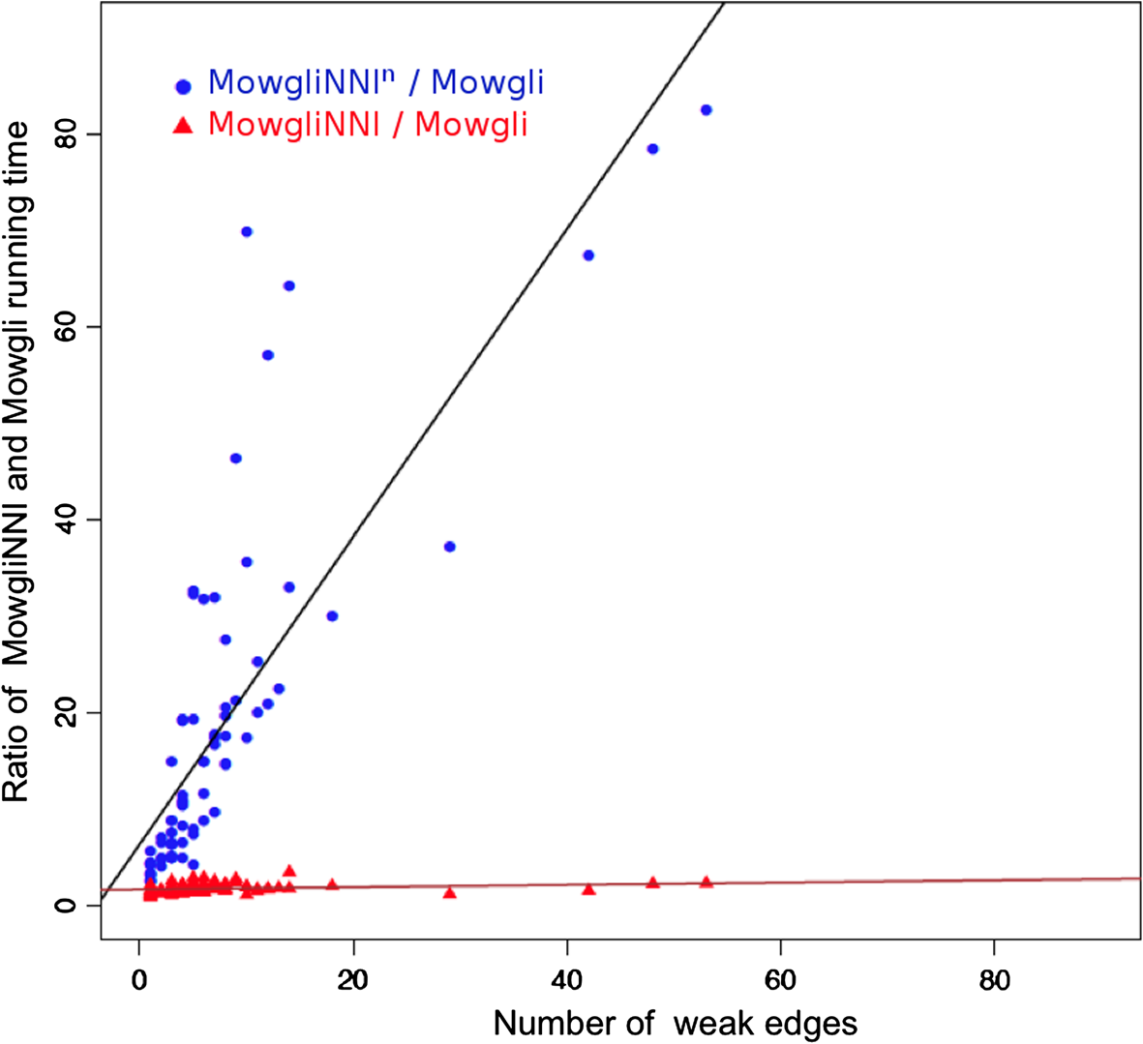
**Compared running times of reconciliation methods.** Two sets of values are plotted: each blue dot, resp. red triangle, corresponds to the ratio between the running time of *MowgliNNI*^*n*^, resp. *MowgliNNI* and that of *Mowgli* on a same gene tree, depending on the number of weak edges. Regression lines are provided for both dot sets.

We measured the running time of *Mowgli* and that of both the naive and optimized versions of *MowgliNNI* (see Methods), respectively called *MowgliNNI*^*n*^ and *MowgliNNI*. From the ≈30*k* families of our dataset, we extracted a random sample of 100 families uniformly spanning from 10 to 80 taxa, and respecting the previously observed proportion of improving / non-improving cases in the reconciliation cost (*i.e.*, 24% and 76% resp.). This sample was used to run the three programs. Figure 13 reports the ratios of *MowgliNNI*^*n*^ and *MowgliNNI* running times over those of *Mowgli*, with respect to the number of weak edges within the input gene tree. Indeed, for each gene tree, the number of tested rearrangements during the optimization search depends on the number of weak edges, which hence strongly impacts *MowgliNNI*^*n*^ and *MowgliNNI* running times. Note that the number of weak edges often represents between 10% and 20% of the gene tree edges, but can go up to 70%.

Figure 13 shows that *MowgliNNI* is 20 (resp. 50 and 80) times faster than *MowgliNNI*^*n*^, when facing 1–20 (resp. 20–40 and 40–60) weak edges. This shows that the combinatorial optimization proposed in the Methods section is crucial in practice.

Now, when compared to *Mowgli*, the rearrangements tried by *MowgliNNI*^*n*^ on weak edges to obtain a better gene tree are done at the price of a relatively small computation time overcost. We also indicate the regression line of *MowgliNNI* running times with respect to those of *Mowgli*, plotted against the number of weak edges. Its slope is only 0.01186, meaning that *MowgliNNI* (the optimized version) is able to take into account the gene tree uncertainties with just a slight increase in the running time.

#### ***Transfers in prokaryotic phyla***

On our whole dataset of 29,709 homologous gene families, we particularly studied transfers in 5 bacterial and 1 archaeal phyla: Proteobacteria (169 genomes), Actinobacteria (31 genomes), Cyanobacteria (14 genomes), Chlamydiae (7 genomes), Spirochaetes (7 genomes) and Crenarchaeota (10 genomes). We compared our results obtained with *Mowgli* and *MowgliNNI* to those of Abby et al. [30] obtained with the Prunier method [29] that infers transfers in mono-copy gene families on another basis than reconciliation.

In order to compare our results to the Abby et al. study, we extracted particular families from HOGENOM v4. For each of the 6 phyla of interest, we collected the list of families having at most one copy of the gene for the genomes of this phylum and separated them into two groups: families having one copy of the gene for each genome of the phylum, so-called “universal families”, and families having a copy of the gene for at least 7 genomes of the phylum, so-called “non-universal families”.

For each phylum, we computed the number of intra-phylum transfers inferred by reconciliations of *Mowgli* and *MowgliNNI* for families of the two groups (universal and non-universal). As the number of families we found in several groups among the various phyla varied slightly from those reported by Abby et al. [30] we summarized the findings of both studies in terms of transfer rates, expressed in number of transfers per million year and per family.

**Figure 14:**
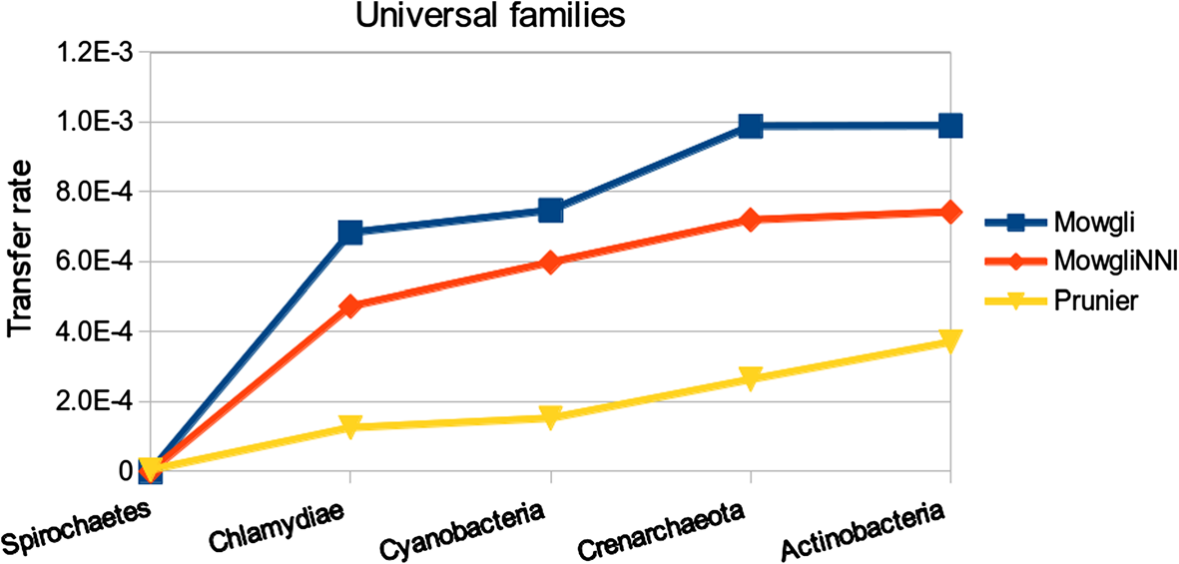
**Impact of *MowgliNNI *on transfer rate inferred on a real dataset of “universal families”.** Comparison of *Mowgli*, *MowgliNNI* and Prunier [29] on the basis of their transfer rate per million year per gene family, for prokaryotic phyla having mono-copy universal families (i.e. families having one copy of the gene for each genome of the considered phylum). No mono-copy universal family was found for Proteobacteria.

**Figure 15:**
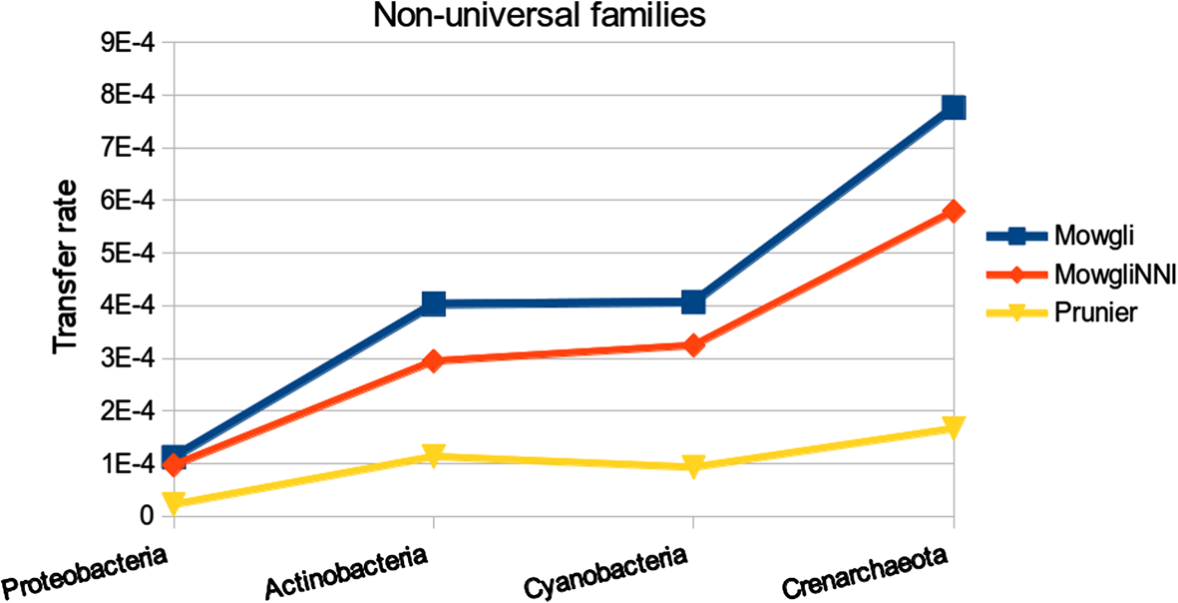
**Impact of *MowgliNNI *on transfer rate inferred on a real dataset of “non-universal families”.** Comparison of *Mowgli*, *MowgliNNI* and Prunier [29] on the basis of their transfer rate per million year per gene family, for prokaryotic phyla having mono-copy non-universal families (i.e. families having at most one copy of the gene for the genomes of a considered phylum, and covering at least 7 genomes of this phylum). No mono-copy non-universal families was found for the Chlamydiae and Spirochaetes phyla.

Figures 14 and 15 display transfer rates of universal, resp. non-universal families, for the studied phyla ordered by increasing transfer rate. We note that the obtained order on the phyla depends on the profile of the studied families, as in Abby et al. [30]. For both the universal and the non-universal families, the transfer rates obtained through *Mowgli* and *MowgliNNI* follow the same trend as that prediced by the Prunier method, i.e. the phyla are ordered in the same way, from the Spirochaetes up to the Actinobacteria in the case of universal families and from the Proteobacteria up to the Crenarchaeota in the case of non-universal families.

Finally, as expected, *MowgliNNI* reduced the number of inferred transfers compared to *Mowgli*, leading to transfer rates closer to that inferred by Prunier.

## Conclusion

We introduce the *MowgliNNI* heuristic method relying on NNI rearrangements of the uncertain parts of the gene trees to solve a parsimony optimization problem for reconciliations accounting for duplications (**), losses (**) and transfers (). We show experimental evidence that reconciliations computed under the parsimony criterion can efficiently correct erroneous parts of gene trees inferred from sequence data. On simulated data, *MowgliNNI* often proposes a new gene tree topology that is closer to the correct one and that also leads to better, and predictions. Moreover, the number of events and the number of most parsimonious reconciliations predicted by *MowgliNNI* are significantly lower than those obtained without questioning the gene tree topology. This is confirmed on real data. A critical point for parsimony methods is the choice of respective costs for the considered evolutionary events. We show here that *MowgliNNI*’s performance is only slightly altered when changing the costs given to the individual events (, and), that is, the method is robust to cost misspecification.

## Appendix

### Proof of Theorem 1

#### 

***Theorem 1.** Consider a gene tree G, the subdivision S*^′^ *of a species tree S, an edge (w, v) of G, a gene tree G*^′^ *obtained by an NNI operation on (w,v), and any strict ancestor u of w in G where the unique child of u that is an ancestor of w is u*_1_ *w.l.o.g. (i.e. w ≤ u*_1_ *in both G and G’). If c(u*_1_ *,x) ≤ ;c*^′^ *(u*_1_*,x) holds for all x ∈ V(S*^′^*), then c (u,x) ≤ c*^′^ *(u,x) holds for all x ∈ V(S*^′^*), and as a corollary C(G,S*^′^*) ≤ C(G*^′^*,S*^′^*).*

#### 

*Proof.* First remark that an NNI operation performed around the edge (*w*,*v*) of *G* to obtain a modified tree *G*^′^ does not alter the order of the nodes above v, which are then considered below indifferently of the tree they belong.

The proof is done with a recurrence over increasing time *t* ∈ {0,1,…,*h*(*r*(*S*^′^))} for the subset of nodes *V*_*t*_(*S*^′^) ⊂ *V*(*S*^′^). Recall that, in *S*^′^ the height of a node *u* (denoted *h*(*u*)) is a valid time function (see Figure 1) and that *u*_1_ is the child of *u* that is an ancestor of *w* (whereas *u*_2_ is incomparable with *w*). □

#### ***Base case***

For time *t* = 0, the possible events for the internal node *u* and any leaf *x* ∈ *V*_0_(*S*^′^) are**,**, andTL (see the reconciliation model of Definition 1).

For each eventE∈{D,T},$c_{E}(u,x)$ (resp.$c_{E}^{\prime }(u,x)$) depends on the costs *c*(*u*_*i*_,*y*) (resp. *c*^′^(*u*_*i*_,*y*)) over all children *u*_*i*_ ∈ {*u*_1_,*u*_2_} and vertices *y* ∈ *V*_*t*_(*S*^′^) (see Definition 3). Since *u*_2_ (resp. *u*_1_) is incomparable to (resp. an ancestor of) *w*, Lemma 2 implies that *c*(*u*_2_,*y*) = *c*^′^ (*u*_2_,*y*) and the assumption states that *c*(*u*_1_,*y*) ≤ *c*^′^(*u*_1_,*y*).

That all costs used in the equation of$c_{E}^{\prime }(u,x)$ are not lower than the corresponding costs in that of$c_{E}(u,x)$ leads to properties listed in the following remark.

##### 

**Remark 1.** The following properties hold for all internal nodes *u* ∈ *V*(*G*)∖*L*(*G*). 

1. For all eventsE∈{D,T} and leaves *x*∈*V*_0_(*S*^′^),$c_{E}(u,x)\leq c_{E}^{\prime }(u,x)$ holds.

2. For all leaves leaves *x* ∈ *V*_0_(*S*^′^),$\text{min}\{c_{E}(u,x):E\in \{D,T\}\}\leq \text{min}\{\overset{\prime }{\underset{E}{c}}(u,x):E\in \{D,T\}\}$.

3. $\underset{x\in V_{0}(S^{\prime })}{\text{min}}c_{\bar{TL}}(u,x)\leq \underset{x^{\prime }\in V_{0}(S^{\prime })}{\text{min}}c_{\bar{TL}}^{\prime }(u,x^{\prime })$, since///SL are impossible events at height 0.

For aTL event of node *u* on a leaf *x* ∈ *V*_0_(*S*^′^), we have the following:

$$\begin{array}{lll} c_{TL}(u,x)\; & =\;\tau +\lambda +\;(\text{where}\;y\;\text{min}.\;c_{\bar{TL}}(u,y)\; & \\ & \;c_{\bar{TL}}(u,y)\;\text{over all}\;y\;\in V(S^{\prime })\setminus \{x\}\; & \\ & \;\text{s.t.}\;\theta_{S^{\prime }}(y)=\theta_{S^{\prime }}(x))\; & \\ & \leq \;\tau +\lambda +\;(\text{where}\;y^{\prime }\;\text{min}.\;{c_{\bar{TL}}}^{\prime }(u,y^{\prime })\; & \\ & \;c_{\bar{TL}}^{\prime }(u,y^{\prime })\;\text{over all}\;y^{\prime }\in V(S^{\prime })\setminus \{x\},\; & \\ & \;\text{s.t.}\;\theta_{S^{\prime }}(y)=\theta_{S^{\prime }}(x))\; & \\ & \;\text{Remark}\; & \\ & =\;c_{TL}^{\prime }(u,x)\;(\text{Definition}\;3)\; & \end{array}$$

Hence, we have the following result:

##### 

**Remark 2.** For all internal nodes *u* ∈ *V*(*G*) ∖ *L*(*G*) and leaves *x* ∈ *V*_0_(*S*^′^),$c_{TL}(u,x)\leq c_{TL}^{\prime }(u,x)$ holds.

And we obtain the following:

$$\begin{array}{ll} \;c(u,x)\; & \;=\;\text{min}\{\;c_{E}(u,x):\;(\text{Definition}\;3;\{D,T,TL\\ & \;\text{are}\;E\in \{D,T,TL\}\;\}\;\text{the only possibilities})\\ & \;\leq \;\text{min}\{\overset{\prime }{\underset{E}{c}}(u,x):\\ & \;E\in \{D,T,TL\}\;\}\;(\text{Remarks}\;1\;\text{and}\;2)\;\\ & \;=\;c^{\prime }(u,x)\;(\text{Def.}\;3;D,T,TL\;\text{are}\\ & \;\text{the only possibilities}) \end{array}$$

Therefore, *c*(*u*,*x*) ≤ *c*^′^(*u*,*x*) holds for each leaf *x* ∈ *V*_0_(*S*^′^).

#### ***Inductive step***

For a height 0 ≤ *t* < *h*(*S*), we now suppose that the expected property *c*(*u*,*y*) ≤ *c*^′^(*u*,*y*) holds for all vertices *y* ∈ *V*_*t*_(*S*^′^) and prove that it still holds for any vertex *x*∈*V*_*t*+1_(*S*).,,,,SL, andTL are the possible events for node *u* and vertex *x*. Following exactly the same arguments as in the base case, *Remark 1* ( and) and *Remark 2* (TL) still hold for the current time (*t*+1).

The dependencies of the corresponding cost for,, andSL events are as follows:$c_{S}(u,x)$ depends on the costs *c*(*u*_*i*_,*x*_*i*_) for *u*_*i*_ ∈ {*u*_1_,*u*_2_} and *x*_*i*_ ∈ {*x*_1_,*x*_2_}, with *x*_*i*_ ∈ *V*_*t*_(*S*^′^);$c_{\emptyset }(u,x)$ on *c*(*u*,*x*_1_), with *x*_1_ ∈ *V*_*t*_(*S*^′^); and$c_{SL}(u,x)$ on *c*(*u*,*x*_*i*_) for *x*_*i*_ ∈ {*x*_1_,*x*_2_}, with *x*_*i*_ ∈ *V*_*t*_(*S*^′^). The same dependencies apply for$c_{S}^{\prime }(u,x)$,$c_{\emptyset }^{\prime }(u,x)$, and$c_{SL}^{\prime }(u,x)$. Recall that *u*_2_ (resp. *u*_1_) is incomparable to (resp. an ancestor of) *w* and that Lemma 2 (resp. the assumption) implies that *c*(*u*_2_,*x*_*i*_) = *c*^′^(*u*_2_,*x*_*i*_) (resp. *c*(*u*_1_,*x*_*i*_) ≤ *c*^′^(*u*_1_,*x*_*i*_)) for each *x*_*i*_ ∈ {*x*_1_,*x*_2_}. Moreover, the inductive hypothesis states that *c*(*u*,*x*_*i*_) ≤ *c*^′^(*u*,*x*_*i*_) holds for each child *x*_*i*_ of *x* since *x*_*i*_ ∈ *V*_*t*_(*S*^′^). For each eventE∈{S,∅,SL}, that all costs used in the equation of$c_{E}^{\prime }(u,x)$ are not lower than the corresponding costs used in the equation of$c_{E}(u,x)$ leads to the following result:

##### 

 For all eventsE∈{S,∅,SL}, internal nodes *u* ∈ *V*(*G*)∖*L*(*G*) and internal vertices *x* ∈ *V*_*t*+1_(*S*^′^),$c_{E}(u,x)\leq c_{E}^{\prime }(u,x)$ holds.

We obtain the following:

$$\begin{array}{ll} \;c(u,x) & =\text{min}\{\;c_{E}(u,x):E\in \;(\text{Def. 3,}\;C\;\text{is not an}\\ & \;\{D,T,TL,S,\emptyset ,SL\}\;\}\;\text{event for}\;u\;\notin \;L(G))\\ & \leq \text{min}\{\;\overset{\prime }{\underset{E}{c}}(u,x):E\in \\ & \;\{D,T,TL,S,\emptyset ,SL\}\;\}\;(\text{Remarks}\;\\ & =c^{\prime }(u,x)\;(\mathrm{Def.}\;C\;\text{is not an}\\ & \;\text{event for}\;u\notin L(G)) \end{array}$$

Therefore, *c*(*u*,*x*) ≤ *c*^′^(*u*,*x*) holds for each vertex *x* ∈ *V*_*t*+1_(*S*^′^), and thus for all vertices of *S*^′^.

As a corollary, the same inequality holds between the root nodes *r* of *G* and *G*^′^, since *w* ≤ *r*. Then *C*(*G*,*S*^′^) ≤ *C*(*G*^′^,*S*^′^).

## Competing interests

The authors declare that they have no competing interests.

## Authors’ contributions

THN, VR, JPD and VB designed the algorithm and the simulation procedure. THN implemented the program and conducted the experiments on the simulated datasets. SP, AAC and VB carried out the experiments on the real dataset. THN, VR, JPD, AAC and VB wrote the paper. All authors read and approved the final version of this manuscript.
